# Novel computational and drug design strategies for inhibition of human papillomavirus-associated cervical cancer and DNA polymerase theta receptor by Apigenin derivatives

**DOI:** 10.1038/s41598-023-43175-x

**Published:** 2023-10-02

**Authors:** Shopnil Akash, Imren Bayıl, Md. Saddam Hossain, Md. Rezaul Islam, Md. Eram Hosen, Amare Bitew Mekonnen, Hiba-Allah Nafidi, Yousef A. Bin Jardan, Mohammed Bourhia, Talha Bin Emran

**Affiliations:** 1 Department of Pharmacy, Faculty of Allied Health Sciences, Daffodil International University, Birulia, Ashulia, Dhaka, 1216 Bangladesh; 2 Department of Bioinformatics and Computational Biology, Gaziantep University, Gaziantep, Turkey; 3 Department of Biomedical Engineering, Faculty of Engineering & Technology, Islamic University, Kushtia, Bangladesh; 4 Professor Joarder DNA and Chromosome Research Laboratory, Department of Genetic Engineering and Biotechnology, University of Rajshahi, Rajshahi, 6205 Bangladesh; 5 Department of Biology, Bahir Dar University, P.O.Box 79, Bahir Dar, Ethiopia; 6 Department of Food Science, Faculty of Agricultural and Food Sciences, Laval University, 2325, Quebec City, QC G1V 0A6 Canada; 7 Department of Pharmaceutics, College of Pharmacy, King Saud University, Riyadh, Saudi Arabia; 8 Department of Chemistry and Biochemistry, Faculty of Medicine and Pharmacy, Ibn Zohr University, 70000 Laayoune, Morocco; 9 Department of Pathology and Laboratory Medicine, Warren Alpert Medical School & Legorreta Cancer Center, Brown University, Providence, RI 02912 United States

**Keywords:** Biotechnology, Computational biology and bioinformatics, Microbiology

## Abstract

The present study deals with the advanced in-silico analyses of several Apigenin derivatives to explore human papillomavirus-associated cervical cancer and DNA polymerase theta inhibitor properties by molecular docking, molecular dynamics, QSAR, drug-likeness, PCA, a dynamic cross-correlation matrix and quantum calculation properties. The initial literature study revealed the potent antimicrobial and anticancer properties of Apigenin, prompting the selection of its potential derivatives to investigate their abilities as inhibitors of human papillomavirus-associated cervical cancer and DNA polymerase theta. In silico molecular docking was employed to streamline the findings, revealing promising energy-binding interactions between all Apigenin derivatives and the targeted proteins. Notably, Apigenin 4′-O-Rhamnoside and Apigenin-4′-Alpha-l-Rhamnoside demonstrated higher potency against the HPV45 oncoprotein E7 (PDB ID 2EWL), while Apigenin and Apigenin 5-O-Beta-d-Glucopyranoside exhibited significant binding energy against the L1 protein in humans. Similarly, a binding affinity range of − 7.5 kcal/mol to − 8.8 kcal/mol was achieved against DNA polymerase theta, indicating the potential of Apigenin derivatives to inhibit this enzyme (PDB ID 8E23). This finding was further validated through molecular dynamic simulation for 100 ns, analyzing parameters such as RMSD, RMSF, SASA, H-bond, and RoG profiles. The results demonstrated the stability of the selected compounds during the simulation. After passing the stability testing, the compounds underwent screening for ADMET, pharmacokinetics, and drug-likeness properties, fulfilling all the necessary criteria. QSAR, PCA, dynamic cross-correlation matrix, and quantum calculations were conducted, yielding satisfactory outcomes. Since this study utilized in silico computational approaches and obtained outstanding results, further validation is crucial. Therefore, additional wet-lab experiments should be conducted under in vivo and in vitro conditions to confirm the findings.

## Introduction

Cervical cancer contributed to 604,127 cases (6.5% of all cancer cases) each year. It inflicted 341,831 deaths (7.7% of all cancer deaths) in 2020 globally, placing it as the fourth most lethal type of cancer in the female population^1^. The worldwide cancer burden is quickly growing as a consequence of continuing demographic and epidemiological shifts, and it is anticipated that this will contribute to a large percentage (> 4,74,000) of deaths among women by the year 2030^2^. Cervical cancer is primarily spread between affluent and less affluent nations, as evidenced by this worldwide cancer burden. evidence shows that viral infections are responsible for 15–20% of all human malignancies. Different phases of cancer development may be accelerated by infection with oncogenic viruses. There are many other forms of Human Papilloma Virus (HPV), but around 15 have been related to cancer. Even if screening procedures are very successful, cervical cancer is still a significant public health issue^3^.

HPV is a small, non-enveloped, icosahedral, double-stranded DNA virus that may transmit through sexual activities. It infects different parts of the body’s organs, such as squamous epithelia, including the skin and upper respiratory and anogenital tract mucous membranes. About 100 various other forms of HPV and around 40 of them are known to infect the anogenital region^4^. Besides, it has been associated with several different malignancies, the most significant of which is cervical cancer. It is also reported that infection with HPV is the root cause of almost all cases of cervical cancer. The occurrence of cervical cancer has been linked to 18 different kinds of human papillomavirus, with types HPV-16 and HPV-18, in particular, being considered high-risk/oncogenic. Low-risk/non-oncogenic HPV strains cause genital warts, especially types HPV-6 and HPV-11. Within a year to two years after infection, cell-mediated immunity typically clears or suppresses most cervical HPV infections^5^.

The HPV may often be confirmed in clinical specimens using hybrid capture or polymerase chain reaction of HPV genomes^6^. HPV genotyping, on the other hand, is accomplished through hybridization with type-specific oligonucleotide probes. All probe assays rely on identifying target nucleic acid patterns by complementing probe nucleic acid patterns that may be replicated through PCR^7^. This identification can be accomplished in a variety of ways. The target DNA is subjected to several cycles of denaturation, primer hybridization, and primer extension in the PCR, which results in the specific to an individual of the target DNA. After 30 cycles of PCR, a proportion of target DNA equal to or more than one million duplicates is created. After that, either a standard dot blot or a Southern blot is used to determine the results of the amplified DNA. One sort of test that uses a modulation scheme to identify DNA or RNA substrates is called a hybrid capture technique^8^. The Hybrid capture method employs a hybridization solution consisting of RNA tags with DNA targets for HPV identification. This is complemented by an immunologically oriented back-end test comparable to an ELISA. Regarding the detection of HPV, the PCR approach has a better analytic sensitivity than the hybrid capture approach; nevertheless, the hybrid capture method may be more efficient in determining women who have concomitant squamous epithelial tumors^9^.

Secondly, a particular kind of genetic recombination known as homologous recombination (HR) occurs when two identical or comparable double-stranded or single-stranded nucleic acid molecules exchange genetic information. The biological system accurately repairs DNA breaks (both strands) through HR where a specific process known as homologous recombinational repair (HRR) contributes^10^. HR deficiency has emerged as a crucial biomarker for multiple types of cancers such as ovarian, breast, pancreatic, and prostate^11^. Mutation in two particular genes, BRCA1 and BRCA2 (combinedly addressed as BRCA1/2), is responsible for HR-mediated DNA repair deficiency leading to the development and initiation of several types of tumors^12^. Interestingly, Poly (ADP-ribose) polymerase inhibitors (PARPi) have shown excellent sensitivity against BRCA1/2-mutated tumors, and many PARPi has been approved in recent decades for clinical use^13,14^. But the main challenge to these medications’ clinical success in patients with HR-deficient tumors is the rapid development of resistance. Therefore, across numerous therapeutic situations, including primary chemotherapy, neoadjuvant treatment, and combination therapy with immunotherapies, the effectiveness of PARPi is now being assessed^15–18^.

Recent research has suggested the synthetic lethality of HR deficiency with DNA polymerase theta making an emerging novel drug target for treating HR-deficient tumors^19^. The DNA polymerase theta contains three domains (N-terminus containing a helicase-like ATPase domain, central domain, and C terminus containing a nuclease domain) and a nuclease domain). It differs from other polymerases in structure and function as it suppresses mitotic crossovers for preserving genomic integrity^20–22^. Any patients suffering from breast and ovarian tumors with HR deficiency have high expression of DNA polymerase theta, which is a backup HR mediator in the DNA double-strand break repair process^23^. As DNA polymerase theta is synthetic and lethal with HR, inhibition of DNA polymerase theta in patients with defective HR can induce tumor cell death^23,24^.

Moreover, the DNA polymerase theta inhibition approach harmonizes with PARPi activity in HR-deficient tumor elimination^23,24^. Synthetic lethality among HR deficiency and DNA polymerase theta inhibition depends on several mechanisms through which DNA polymerase theta operates to keep the genome stable and stop tumorigenesis^21^. Besides, the DNA polymerase theta is critical in mutagenic microhomology-mediated end-joining (MMEJ), an significant DNA double-strand break-repairing mechanism^25^. However, the mechanism by which the DNA polymerase theta maintains synthetic lethality with HR-deficient tumors remains unclear, but this evidence suggests that the versatile functionality of the DNA polymerase theta is vital for HR-deficient tumor survival. However, The DNA polymerase theta demonstrates distinct characteristics of drug ability, offering a compelling case for creating DNA polymerase theta inhibitors^26^. Therefore, developing an inhibitor targeting DNA polymerase theta should be a rational option for curing HR-deficient tumor cells.

There is currently no therapy available for HPV^27^. So, potential treatment or drug is highly needed to manage HPV and its related cancer. But, developing an effective medication with a high degree of potentiality is a very time-consuming matter, and required huge research funds. Besides, during the developing phases, many drugs fail, and can’t go final stages due to unwanted effects^28^. Resulting, the research community may lose huge amounts of resources and costs. But, in the modern era of drug development, this huge cost could be minimized by early investigation of physiochemical, and toxicity prediction^29^. So, in this investigation, the most popular in silico application, and investigation are applied and determined the drug-like properties of Apigenin derivatives for the treatment of human papillomavirus, and its associated cancer.

Here, we also performed a comprehensive computational investigation supported by a rigorous literature-based approach aiming to identify the most potent DNA polymerase theta inhibitor from selected Apigenin derivatives. These Apigenin derivatives were evaluated based on their ability to inhibit DNA polymerase theta ATPase activity and prevent the MMEJ repairing mechanism which will induce HR-deficient tumor cell death.

## Literature studies and ligand-receptor selection criteria

### Genomic structure of HPV

The papillomavirus genome comprises three distinct sections and is formed of a tiny, double-stranded, highly conserved DNA that is around 8000 base pairs in length. Understanding the molecular biology of this small DNA molecule is complicated. There are seven proteins totaling 4000 base pairs (bp) that are involved in transcription and replication and cell metamorphosis; they include six early proteins, three regulatory proteins (E1, E2, and E4), and three oncoproteins (E5, E6, and E7). The viral capsid is composed of two proteins, L1 and L2, encoded by a separate 3,000 bp section of DNA. A 1000 bp section called the long control region (LCR) encodes the viral DNA replication and transcriptional regulatory components. The L1 protein is necessary for the development of viral pathogenicity, and it is responsible for the promotion of virion attachment to heparin sulfate receptors in the basal membrane^30^. Again, the E1 protein forms a hexameric complex that attracts topoisomerase I, DNA polymerase, and replication protein A (RPA), all of which are required for viral replication^31^. The E1 protein also urges DNA breaks in host chromatin, which aids viral integration, and the E2 transcription factor regulates the E6 and E7 ORFs. When abundant, E2 binds to the 5′-ACCG(N)4CGGT-3′ palindromic sequence found in E2 binding sites (E2BS) in LCR, including the P97 promoter^32^. The E4 protein, the most expressed viral protein, is described in suprabasal and granular epithelial layers. The E4 interacts with keratin-associated amyloid fibers, causing cell fragility and contributing to virion release^33^.

### Development of HPV in cervical cancer

The process of cervical cancerogenesis, in which HPV gene integration occurs between other cellular alterations and epigenetic factors, is a complicated mechanism of unregulated cellular proliferation. Mutations in the DNA caused by the cell’s environment and HPV infection may allow the virus' DNA to integrate with the host’s DNA synthesis machinery and cause replication of the virus. Thus, the virus can bypass cellular and immunological defenses while encouraging cell growth and blocking apoptosis^3^. The Two primary oncogenic protein products E6 and E7 control the cell cycle by maintaining the process of normal apoptosis and they play an essential role in promoting oncogenesis in cells. By duplicating their genetic material (DNA), viruses may produce cells to exhibit characteristics of cancer, including uncontrol growth, angiogenesis, invasion, metastasis, and resistance to apoptosis and growth suppressors^34^.

### Role of DNA polymerase theta in disease development

DNA polymerase theta is a family of DNA polymerases (Pol θ) that has been essential to maintaining DNA repair and damage tolerance. When any problematic condition occurs in the double strand of DNA, it helps to repair it. Some studies have reported that when the DNA polymerase theta is overexpressed in cancer cells, it may promote the resistance of chemotherapeutic or cancerous agents. As a result, it makes it difficult to treat cancer. So, the DNA polymerase theta might be a potential target receptor for the discovery of a drug to treat breast and ovarian cancers, etc.^35,36^.

### Apigenin pharmacological evidence

Apigenin is a flavonoid that may be found in a wide variety of fruits, vegetables, and plants used in traditional Chinese Medicine. This multifunctional molecule has several different biological functions, including anti-inflammatory, antioxidant, antibacterial, and antiviral properties. As a result, Apigenin has a long history of usage in the context of alternative and conventional medical practices. Apigenin has been connected to having an antitumor effect against a broad spectrum of cancers. This effect is thought to be achieved through apoptosis and autophagy stimulation, cell cycle arrest, inhibition of cell migration and invasion, and an increase in immunological response^37,38^, and it may demonstrated to be capable of preventing, inhibiting, or reversing the effects of chemically induced genotoxicity in vitro cell models, in vivo investigations, and AMES tests employing bacterial models. This anti-mutagenic effect has been demonstrated and proven. The anti-carcinogenic function of Apigenin has received a lot of attention recently and has been discovered to be protective against different kinds of cancer, including breast cancer, cervical cancer, prostate cancer, skin cancer, thyroid cancer, colon cancer, leukemia, lung cancer, endometrial cancer, neuroblastoma, and adrenocortical cancer^39,40^. As Apigenin is composed of a multifunctional role against a number of diseases. So, this compound is chosen in our current computational experiment as a target biomolecule.

## Method and material

### Ligand preparation and molecular optimization

**Figure 1:**
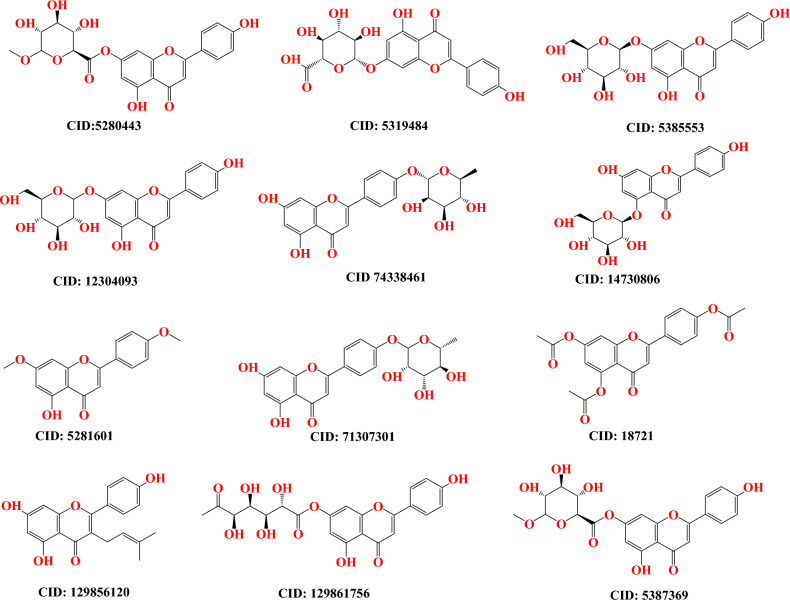
Chemical structures of studied compounds.

The technique of optimization was worked out to achieve the best possible outcomes with regard to the performance of the molecular docking approach as well as the arrangement of molecules in a three-dimensional framework. In the outset, a three-dimensional framework of Apigenin analogs was retrieved by obtaining from the PubChem database (https://pubchem.ncbi.nlm.nih.gov/) (Fig. 1)^41^. This allowed for the structure to be observed in three dimensions.

Prior to molecular docking, the ligands were optimized using the Gaussian09 software with the DFT/B3LYP-6311G method. However, no aqueous operation optimization was conducted as it is not necessary for ligand optimization. The purpose of these optimizations was to prepare the ligands for molecular docking. The molecular docking method was employed to select poses with the highest binding affinity (measured in kcal/mol) between apigenin and apigenin 5-O-Beta-D Glucopyranoside ligands and the L1 protein of human papillomavirus (PDB ID 6L31). Subsequently, the best-selected poses underwent molecular dynamics simulation. No separate ligand optimization was performed for the ligands in both complex structures. The topology files of the complex structures were prepared using Charmm-gui, eliminating the need for a separate optimization for the ligands.

### Protein preparation and molecular docking study

**Figure 2:**
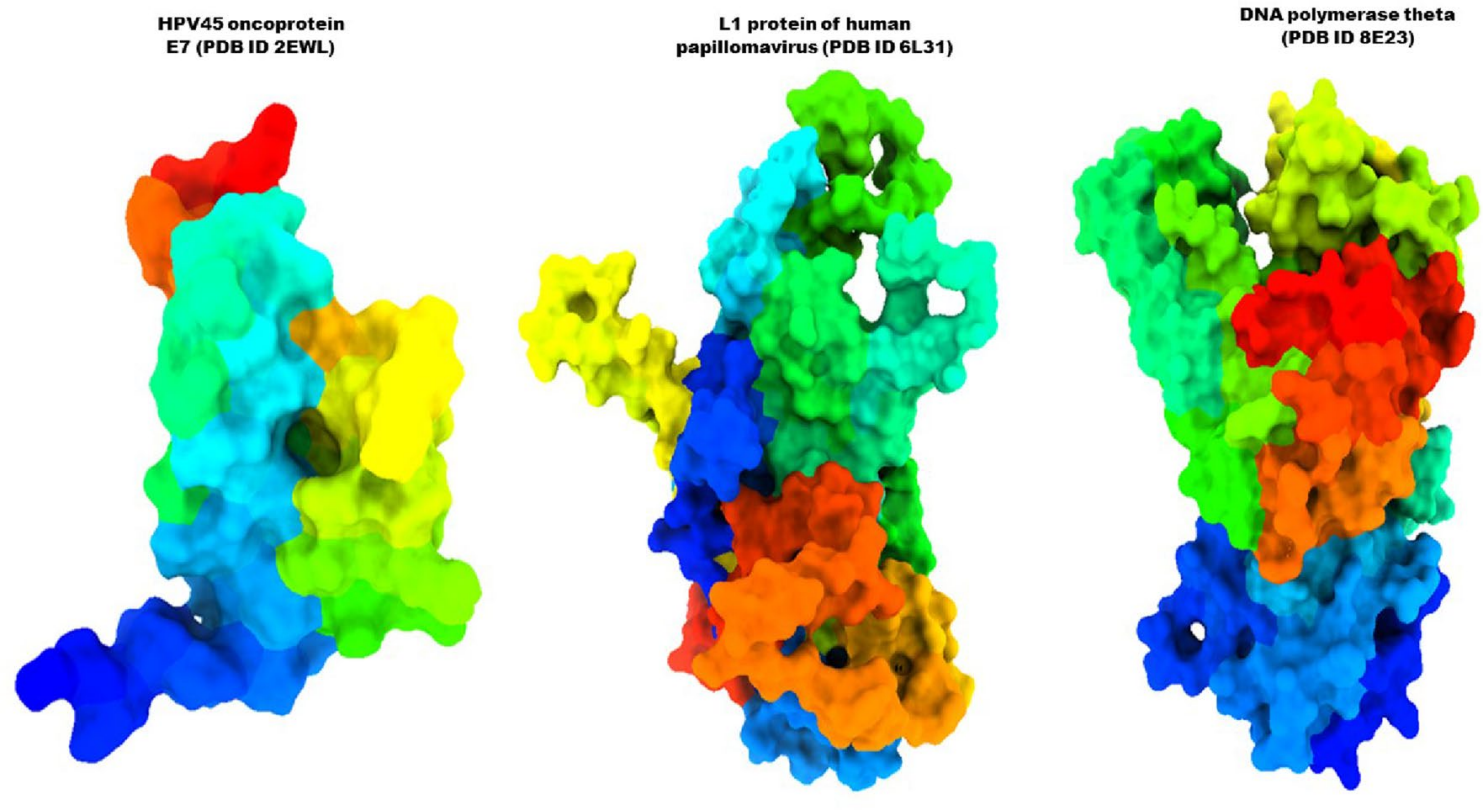
Three-dimensional protein structure of the targeted receptor.

The primary objective of the molecular docking technique is to make an informed prediction regarding the composition of the ligand-receptor complex through the utilization of computational approaches. The docking process encompasses two distinct yet interdependent steps. Firstly, it involves sampling several conformations that the ligand can adopt when bound to the protein’s active site. Secondly, it entails the classification of these residues based on a performance index. By executing these intricately woven steps, molecular docking sheds between ligands and receptors are performed, and unraveling the prediction of their interactions and aiding drug discovery and design endeavors^42^. The PyRx application was employed to accomplish molecular docking^43^. Before that, the crystal structure of HPV45 oncoprotein E7 (PDB ID 2EWL), the L1 protein of human papillomavirus (PDB ID 6L31), and DNA polymerase theta (PDB ID 8E23) were collected from the RCSB protein data bank^36,44,45^. All of the crystal solvent constituents were removed, together with the native agonist and any extra compounds from the crystal structure, and prepared for docking study (Fig. 2 showing the targeted structure). During molecular docking studies, the grid box coordinates were strategically set to cover both the entire protein and the site of interest, ensuring accurate ligand placement. The grid center points were set to X = − 34.66, Y = − 18.07, Z = 23.3766, and the box dimensions (Å) X = 26.45, Y = 53.3637, Z = 39.77 for (PDB-ID: 2EWL), the grid center for (PDB ID 6L31); X = − 11.279, Y = − 28.1119, Z = − 28.2088, and box dimension X = 78.2212, Y = 22.88 and Z = 87.982 and the grid center for (PDB ID 8E23); X = − 19.552, Y = − 26.86772, Z = − 34.998, and box dimension X = 80.99, Y = 27.99 and Z = 83.112 were set so that the grid box could wrap the whole substrate binding pocket of the protein structure.

### Determination of ADMET, and pharmacokinetics, and drug-likeness

During the stage of drug discovery and development, it is completely obvious that the pharmacokinetic (PK) characteristics of potential therapies, and more specifically ADMET (absorption, distribution, metabolism, excretion, and toxicity), are critical factors to consider^46^. Consequently, the Apigenin derivatives that were being explored were initially placed through a critical drug-likeness screening using the SwissADME and pkCSM webservers^47^. This testing was based on calculated physicochemical and ADMET-related parameters. The SMILES strings of the ligands that were utilized as the input for the molecular markers for both websites. In point of fact, unfavorable pharmacokinetic features of potential drug candidates are a significant contributor to the rate of failure throughout clinical trials^48^. Because of this, it is vital to conduct pre-clinical evaluations of the pharmacokinetic characteristics and drug-likeness of proposed medications.

### Molecular dynamics simulation (MDs) protocol

To understand the behavioral changes that occur in the protein–ligand complex when it is exposed to a dynamic environment on an atomic level, MD is a good computer simulation method currently receiving a lot of attention in drug development research^49^. It is also an indispensable instrument for determining the intra- or interatomic interaction stability of the protein–ligand complex over a user-specified period^50^. The best-scoring docking models of the most potential Apigenin, and Apigenin-5-O-beta-D-glucopyranoside, apo form L1 protein of human papillomavirus (PDB ID 6L31), were chosen as the starting points for a 100-ns all-atom molecular dynamics simulation using the GROMACS-2019 software (GNU, General Public License; http://www.gromacs.org). The CHARMM36 force field and the Gromacs version 2019 software package were employed to conduct simulations running for 100 ns within a periodic water box^51,52^. The force field for Apigenin and Apigenin-5-O-beta-D-glucopyranoside, as well as the apo form L1 protein of human papillomavirus, was generated using the CHARMM-GUI server. Each complex was placed inside a rectangular box with a buffer distance of 10 in each direction and solvated with TIP3P water molecules. To neutralize the system’s charge for the Apigenin ligand, 5 Na + ions and 0 Cl– ions were added. Similarly, 5 Na + ions and 0 Cl– ions were added to neutralize the system’s charge for the Apigenin-5-O-beta-d-glucopyranoside ligand, while 33 Na + ions and 0 Cl– ions were added for the L1 protein of human papillomavirus. Following this, 0.0 M of NaCl was added to create an environment similar to cellular conditions for each complex.

The complexes were subjected to structure minimization using the CHARMM36 force field. Each system was then equilibrated at a temperature of 310 K for 5000 steps (10 PS) in the NPT ensemble during the production run, which lasted for 100 s. The position, velocity, and energy of the system were recorded every 10 ps. Hydrogen atoms were constrained using the Lincs technique. A switching method of 12–14 was employed to calculate van der Waals forces, with a cutoff value of 14^53,54^. Long-range electrostatic interactions were calculated using the particle mesh Ewald (PME) approach, with a maximum grid spacing of 1.2. The PME computations were conducted at each step, without the use of a multiple-time stepping scheme. The temperature was maintained at 310 K, and the system size changes in the barostat were targeted at 1 bar. The integration time step was set to 2 fs (0.002 = dt), and nsteps = 50,000,000 (50,000,000*2 = 100,000,000 ps = 100 ns). Post-simulation, the trajectories were analyzed using the VMD (University of Illinois at Urbana-Champaign, Urbana, IL, USA) program, Bio3D, and QTGRACE, respectively, following re-centering of the simulation output^55^. After each MD simulation was completed, its trajectory was analyzed to determine a variety of characteristics, such as the root mean square deviation (RMSD), root mean square fluctuation (RMSF), the radius of gyration (Rg), solvent-accessible surface area (SASA), hydrogen bonds (H-bonds), principal component analysis (PCA), and dynamics cross-correlation map (DCCM). The resulting files were analyzed and visualized using xmgrace (https://plasma-gate.weizmann.ac.il/Grace/), Bio3D, and VMD software^56,57^.

### Binding free energy calculation

Molecular mechanics/Poisson-Boltzmann surface area (M-PBSA) methodology provides a comprehensive analysis of the quantitative assessment of the interaction mechanism between proteins and ligand molecules. The current investigation utilizes the MM-PBSA technique to evaluate the binding affinity of a complex consisting of a protein and a ligand, to obtain a deeper understanding of the fundamental binding mechanism. The calculation of the van der Waals energy, electrostatic energy, polar solvation energy, and binding energy was conducted for the Apigenin and Apigenin 5-O-Beta-D-Glucopyranoside complexes to forecast the overall ΔGbind. The calculation of the binding free energy for the protein–ligand reaction was performed in the following manner: The equation ΔGbind = G complex–(G protein + G ligand) is utilized to determine the total binding energy of the protein–ligand complex, where G protein denotes the binding energy of the protein and G ligand represents the binding energy of Apigenin and Apigenin 5-O-Beta-D-Glucopyranoside, as examined in this investigation.

### Density functional theory (quantum mechanics)

Density functional theory was used to conduct a quantum mechanical calculation on the top twelve compounds (hits) from the virtual screening. The Gaussian 09W program was used for the calculations by optimizing the compounds' geometries at DFT/B3LYP/6-31G (d’p’) levels^58,59^. The compounds were analyzed to determine their electron acceptor and electron donor properties by calculating the frontier orbital energies, including the highest occupied molecular orbital (HOMO) and the lowest occupied molecular orbital (LUMO), as well as the energy gap and molecular electrostatic potential. These characteristics also provide information regarding the chemical reactivity and stability of compounds^60^.

## Results and discussion

### Lipinski rule and pharmacokinetics

The complex balance of all molecular and structural characteristics (molecular weight, lipophilicity, rotatable bonds, surface area, number of hydrogen bond acceptors and donors, bioavailability), as determined by the specific evolution of various computational filters developed by Lipinski rule and it is included in the concept of drug-likeness. Our reported compound has a minimum violation of Lipinski rule except for two compounds (CID 5319,484 and 129861,756) and these compounds have minimum bioavailability scores while others have very good bioavailability scores and most of them are about 0.55 or 55% bioavailability (showing in Table 1).

**Table 1:** Data of lipinski rule, pharmacokinetics.

PubChem CID	Molecular weight	Hydrogen bond acceptor	Hydrogen bond donor	Consensus Log P_o/w_	Lipinski rule		Bioavailability
					Result	violation	
5280443	270.24	5	3	2.11	Yes	0	0.55
5319484	446.36	11	6	0.28	No	2	0.11
5,385,553	432.38	10	6	0.55	Yes	1	0.55
12304093	432.38	10	6	0.55	Yes	1	0.55
74338461	416.38	9	5	1	Yes	0	0.55
14730806	432.38	10	6	0.16	Yes	1	0.55
5281601	298.29	5	1	2.79	Yes	0	0.55
71307301	416.38	9	5	1	Yes	0	0.55
18721	396.35	8	0	2.98	Yes	0	0.55
129856120	338.35	5	3	3.32	Yes	0	0.55
129861756	476.39	12	6	0.13	No	2	0.17
5387369	460.39	11	5	0.82	Yes	1	0.55

### Molecular docking analysis against targeted receptor

Initially, ADMET, PK, and drug-likeness were assessed, and it has been documented that most of the molecules had accepted the guidelines of the Lipinski rule, PK, or the ADMET calculations. As a consequence of this, these compounds were molecularly docked and subjected to further screening. The binding affinity is determined to measure how tightly inhibited or bonded the drugs with targeted protein are during the formation of complex structure^61^. It is said that binding affinities of molecules greater than – 6.0 kcal/mol should be potential drug candidates^62^. After molecular docking, the result has been documented that the majority of the compounds have been shown to have potent interactions and greater binding energies with both target proteins. In Table 2, the most effective compounds’ interactions with the HPV45 oncoprotein E7 (PDB ID 2EWL) protein are reported Apigenin 4’-O-Rhamnoside, and Apigenin-4’-Alpha-L-Rhamnoside along with their docking scores − 6.9 kcal/mol, and − 6.7 kcal/mol against HPV45 oncoprotein E7 (PDB ID 2EWL). Besides, the binding affinities of the L1 protein of human papillomavirus (PDB ID 6L31) ranged from − 7.7 kcal/mol to − 9.3 kcal/mol, whereas molecules Apigenin and Apigenin 5-O-Beta-d-Glucopyranoside showed significant binding energy against L1 protein of human.

**Table 2:** Binding affinity against targeted protein.

Chemical name with PubChem CID	HPV45 oncoprotein E7 (PDB ID 2EWL)	L1 protein of human papillomavirus (PDB ID 6L31)	DNA polymerase theta (PDB ID 8E23)
	Binding affinity (kcal/mol)	Binding affinity (kcal/mol)	Binding affinity (kcal/mol)
Apigenin (CID:5280443)	− 5.7	− 9.1	− 7.7
Apigenin 7 glucuronide (CID: 5319484)	− 6.3	− 8.7	− 7.9
Apigenin 7-O-beta-d-glucoside (CID: 5385553)	− 6.4	− 8.5	− 8.2
Apigenin-7-O-glucoside (CID: 12304093)	− 6.6	− 8.5	− 7.7
Apigenin-4′-Alpha-l-rhamnoside (CID 74338461)	− 6.7	− 8.0	− 8.8
Apigenin 5-O-beta-d-glucopyranoside (CID: 14730806)	− 5.9	− 9.3	− 8.5
Apigenin 7,4’-dimethyl ether (CID: 5281601)	− 5.6	− 8.2	− 7.2
Apigenin 4’-O-rhamnoside (CID: 71307301)	− 6.9	− 8.1	− 8.4
Apigenin triacetate (CID: 18721)	− 6.5	− 8.1	− 7.5
Prenyl apigenin (CID: 129856120)	− 6.0	− 8.6	− 7.9
Apigenin-7-O-methyl glucuronate (CID: 129861756)	− 6.3	− 7.7	− 7.5
Apigenin 7-O-methylglucuronide (CID: 5387369)	− 6.4	− 8.4	− 8.0

Secondly, DNA polymerase plays an essential role in the therapeutic strategy of cancer and some other disease by blocking or inhibiting DNA polymerases. Many hyperproliferative conditions, such as cancer, autoimmune diseases, and viral infections, are treated with drugs that block DNA synthesis. So, the DNA polymerase theta is also included in this investigation, and perform molecular docking to determine the capability of whether the reported Apigenin derivatives can inhibit the DNA polymerase theta or not and how much binding affinity is produced during binding with each other. This time, the binding affinity range is achieved − 7.5 kcal/mol to − 8.8 kcal/mol which represents that mentioned Apigenin derivatives should be inhibited the DNA polymerase theta (PDB ID 8E23). Besides, the vast majority of the Apigenin derivatives revealed more potent interactions and also exhibited strong interactions and optimum binding affinity with the target. So, the Apigenin derivatives suggested performing wet-lab synthesis and then evaluated on a biological or practical value, to establish as potential drug candidates.

### Molecular docking pose and active site analysis

**Figure 3:**
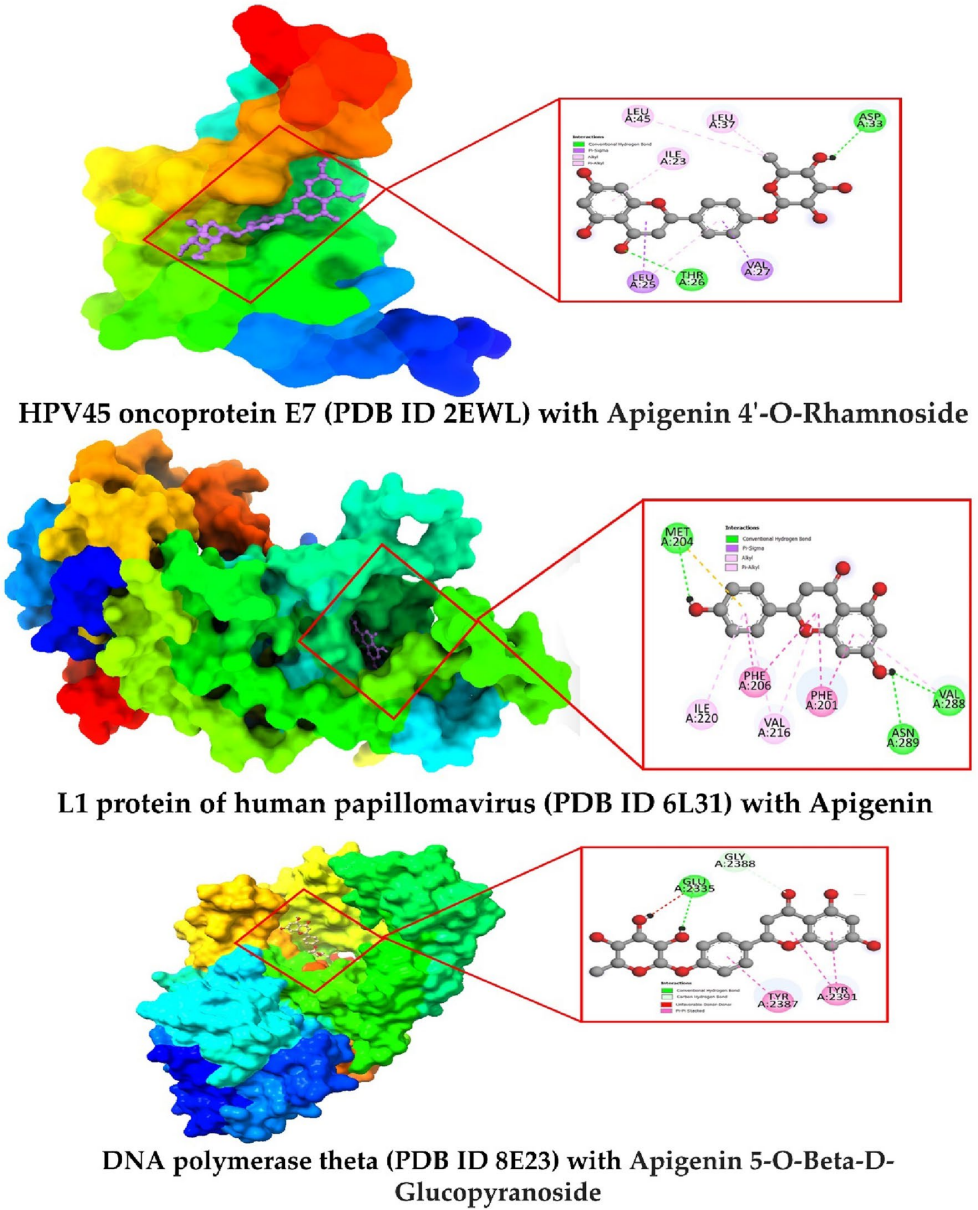
Docking interactions between the proposed compound.

Molecular docking pose and active site analysis has been done by using discovery studio and Chimera X application. It helps to understand and visualize the specific amino acid residue where the ligand binds and formed a drug-protein in the complex (Fig. 3). In this study, the best two complexes are visualized based on maximum binding energy. The first one is drug protein complex of HPV45 oncoprotein E7 (PDB ID 2EWL) with Apigenin 4'-O-Rhamnoside where the active site are formed LEU A:45, LEU A:37, ASP A:33, ILE A: 23, LEU A: 25, THR A: 26, VAL A: 27 LEU A:45, LEU A:37, ASP A:33, ILE A: 23, LEU A: 25, THR A: 26, VAL A: 27. Similarly, the second one is drug protein complex of L1 protein of human papillomavirus (PDB ID 6L31) with Apigenin. This time, the active residue or binding site are located and form MET A:204, ILE A: 220, PHE A: 206, VAL A:216, PHE A: 201, ASN A:289, and VAL A: 268.

### Theoretical ADMET data analysis

The predicted ADMET data of reported compounds are available in Table 3. Few chemicals showed excellent human intestinal absorption and water solubility. This could help substances have an increased blood concentration for the best biological activity. Additionally, these substances showed low blood–brain barrier (BBB) penetration, indicating a free from the creating CNS or neurotoxicity. Most of the reported ligands were not causes of inhibitors of cytochrome P450 enzyme which indicate compounds are perfectly metabolized by cytochrome enzyme which is found in lever.

**Table 3:** ADMET data of reported ligand.

CID	Absorption			Distribution		Metabolism		Excretion		Toxicity		
	Water solubility Log S	Caco-2 permeability × 10^–6^	Human intestinal absorption (%)	VDss (human)	BBB permeability	CYP450 1A2 inhibitor	CYP450 2D6 substrate	Total clearance (ml/min/kg)	Renal OCT2 substrate	AMES toxicity	Skin sensitization	Hepatotoxicity
5280443	− 3.329	1.007	High	0.822	No	Yes	No	0.566	No	No	No	No
5319484	− 2.762	0.693	Low	0.319	No	No	No	0.588	No	No	No	No
5385553	− 2.762	0.33	Low	0.342	No	No	No	0.547	No	No	No	No
12304093	− 2.559	0.33	Low	0.342	No	No	No	0.547	No	No	No	No
74338461	− 3.081	0.488	Low	1.258	No	No	No	0.56	No	No	No	No
14730806	− 2.549	0.418	Low	0.399	No	No	No	0.552	No	No	No	No
5281601	− 3.714	1.106	High	− 0.1	Yes	Yes	No	0.737	Yes	No	No	No
71307301	− 3.081	0.488	Low	1.258	No	No	No	0.56	No	No	No	No
18721	− 4.609	1.322	High	− 0.563	No	Yes	No	1.058	No	No	No	Yes
129856120	− 2.958	0.034	High	− 1.691	No	No	No	1.057	No	No	No	No
129861756	− 2.981	0.052	Low	1.184	No	No	No	0.562	No	No	No	No
5387369	− 3.097	0.204	Low	1.071	No	No	No	0.643	No	No	No	No

Many mechanisms, primarily the liver and kidney, are used to excretion of drug compounds from the body. Smaller drug molecules (< 300) were excreted from bile whereas bigger drug molecules (> 500) were removed from urine. Between 300 and 500 molecular weights are excreted from bile and urine both^63^. For determining the excretion level of drug compounds, the total clearance rate of specific compounds is shown in Table 3. Organic cation transporter 2 or OCT2 is another parameter that help to renal clearance of compounds. Where thus compounds did not expect to substrate on renal OCT2.

Unanticipated drug toxicity is a crucial factor in the failure of successful drug candidates and the withdrawal of marketed treatments. So, toxicity prediction of drug is one of the first requirements for the development and discovery of the drugs. Therefore Table 4 present several toxicity parameters as: AMES toxicity, skin sensitization and hepatotoxicity. In this research reported every article show positive result in ADMET prediction, which are non-toxic drug compound except one hepatotoxic compound.

**Table 4:** Binding energy results.

Compounds name	ΔEVDW (kJ/mol)	ΔEEEL (kJ/mol)	ΔGPB (kJ/mol)	ΔGNP (kJ/mol)	ΔGDISP (kJ/mol)	ΔG Binding (kJ/mol)
Apigenin	− 35.52	− 23.39	32.49	− 3.19	0.0	− 29.61
Apigenin 5-O-Beta-d-Glucopyranoside	− 0.40	0.09	0.29	− 0.11	0.0	− 0.13

### QSAR and pIC50 calculation

Multiple linear regression (MLR) analysis was used in quantitative structure–activity relationship (QSAR) investigations to determine the influence of compounds on pharmacological activity. The relationship between biological activities and structural activities of chemical compounds has been calculated using the quantitative structure activities relationship (QSAR) method. It is discovered that various compounds have distinct QSAR and pIC_50_ values, and the total value of the QSAR and pIC_50_ investigation fits all the requirements. The range of QSAR and pIC50 is determined to be between 5.09 and 4.58, with 5.09 being the higher value and 4.58 being the lower value. According to Table 5, the predicted pIC50 indicates that these described compounds may have biological significance against human papilloma virus. Following equation is applied which developed by another publication^64^.

**Table 5:** Data of QSAR and pIC50 data.

CID	Chiv5	bcutm1	MRVSA9	MRVSA6	PEOEVSA5	GATSv4	J	Diameter	pIC_**50**_
5280443	1.332	4.1	10.969	52.688	0.0	0.874	1.65	10.0	4.59
5319484	2.235	4.105	16.939	52.688	0.0	0.891	1.403	15.0	4.94
5385553	2.244	4.105	10.969	52.688	0.0	0.886	1.394	15.0	5.08
12304093	2.244	4.105	10.969	52.688	0.0	0.886	1.394	15.0	5.08
74338461	2.191	4.102	10.969	52.688	0.0	0.881	1.285	15.0	5.04
14730806	2.293	4.102	10.969	52.688	0.0	0.868	1.436	15.0	5.09
5281601	1.496	4.102	10.969	52.688	0.0	0.824	1.62	12.0	4.78
71307301	2.191	4.102	10.969	52.688	0.0	0.881	1.285	15.0	5.04
18721	1.772	4.108	28.877	52.688	0.0	0.806	1.629	14.0	4.58
129856120	1.883	4.121	10.969	63.834	11.649	0.878	1.771	10.0	4.98
129861756	2.401	4.119	16.939	46.622	0.0	1.028	1.557	13.0	4.85
5387369	2.326	4.105	16.939	52.688	0.0	0.912	1.405	16.0	5.03

Here, $${\text{pIC5}}0 \, \left( {{\text{Activity}}} \right) \, = \, - { 2}.{768483965 } + \, 0.{133928895 } \times \, \left( {{\text{Chiv5}}} \right) \, + { 1}.{59986423 } \times \, \left( {{\text{bcutm1}}} \right) \, + \, \left( { - \, 0.0{23}0{9681}} \right) \, \times \, \left( {{\text{MRVSA9}}} \right) \, + \, \left( { - \, 0.00{29461}0{1}} \right) \, \times \, \left( {{\text{MRVSA6}}} \right) \, + \, \left( {0.00{671218}} \right) \, \times \, \left( {{\text{PEOEVSA5}}} \right) \, + \, \left( { - \, 0.{15963415}} \right) \, \times \, \left( {{\text{GATSv4}}} \right) \, + \, \left( {0.{2}0{7949857}} \right) \, \times \, \left( {\text{J}} \right) \, + \, \left( {0.0{82568569}} \right) \, \times \, \left( {{\text{Diametert}}} \right).$$

### Molecular dynamics simulation analysis

The substantial root mean square deviation (RMSD) value can be attributed to the protein's considerable size and its composition of five distinct chains. During the molecular dynamics’ simulation, an energy minimization procedure was conducted at a time scale of 100 ns. Subsequently, the system was brought to a state of equilibrium. For energy minimization all three system, Using the steepest descent algorithm, the energy of each system was minimized until the maximal force was less than 1,000,000 kj/mol/nm.

This was performed to eliminate any steric conflicts within the system. An isothermal-isochoric ensemble NVT (constant number of particles, volume, and temperature) and an isothermal-isobaric ensemble NPT (constant number of particles, pressure, and temperature) were used to equilibrate each system. At 310 K and 1 bar pressure, the two types of ensemble equilibration methods stabilized the three systems. After the molecular dynamics simulation at 100 ns was completed, the PBC effect was eliminated by using the code gmx trjconv –f step5.xtc –o new.xtc –s step5.tpr –pbc mol –center –n index. ndx–ur compact in the Gromacs software, and then it was checked in the VMD program and it was seen that there was no jump.

#### Root-mean-square deviation (RMSD) analysis

**Figure 4:**
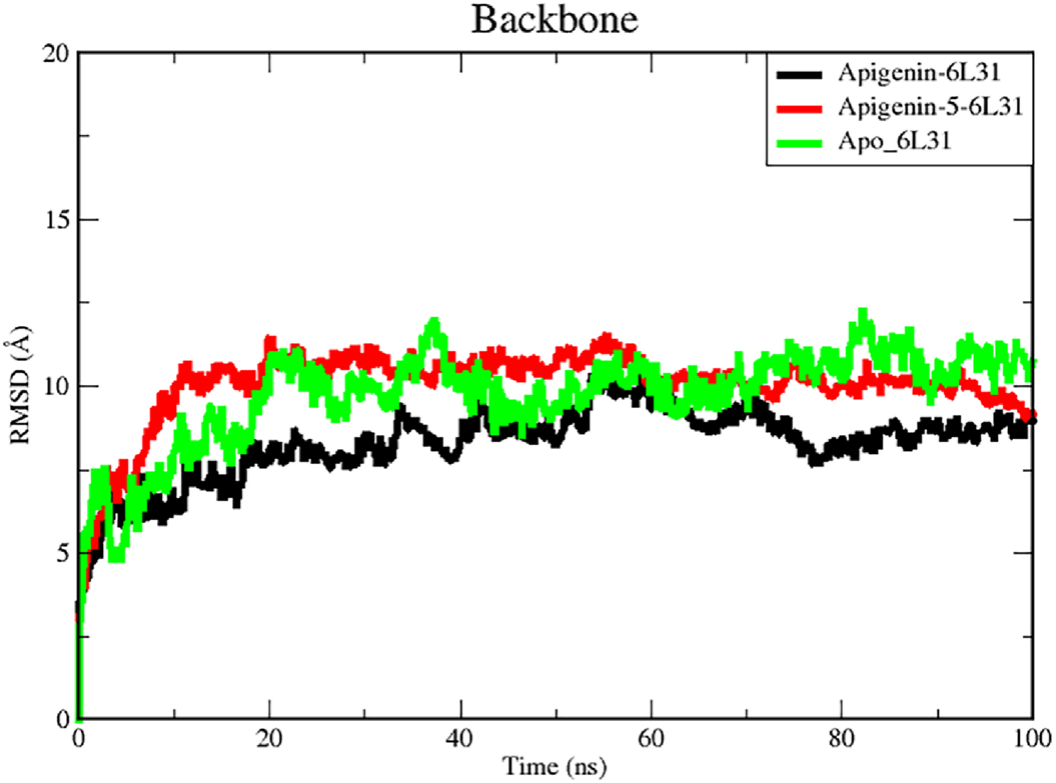
RMSD analysis of Apo and the ligand complex (C-Alpha) in molecular dynamics simulations for the time scale of 100 ns. The black and red colors represented the Apigenin, rand Apigenin-5-O-beta-d glucopyranoside complexes with L1 protein of human papillomavirus (PDB ID 6L31) the green is represented the Apo protein (PDB ID 6L31).

To gain a better understanding of the dynamic behavior and stability, the results of MD simulations for both the apo form and the ligand complex are investigated on a time scale of 100 ns. The MD simulation is carried out for a total of one hundred nanoseconds, and the trajectories for the RMSD plot are displayed in Fig. 4. The colors in the figure are those associated with Apo protein at a time scale of 100 ns when it is complexed with Apigenin and Apigenin-5-O-beta-d-glucopyranoside. The root means square deviation (RMSD) provides an interpretation regarding the extent to which a group of atoms deviates from the appropriate original reference structure of a protein, ligand, or even a ligand–protein complex. A substantial amount of instability, which is related to changes within the conformation of the molecule being researched, can be correlated with having high RMSD values.

**Figure 5:**
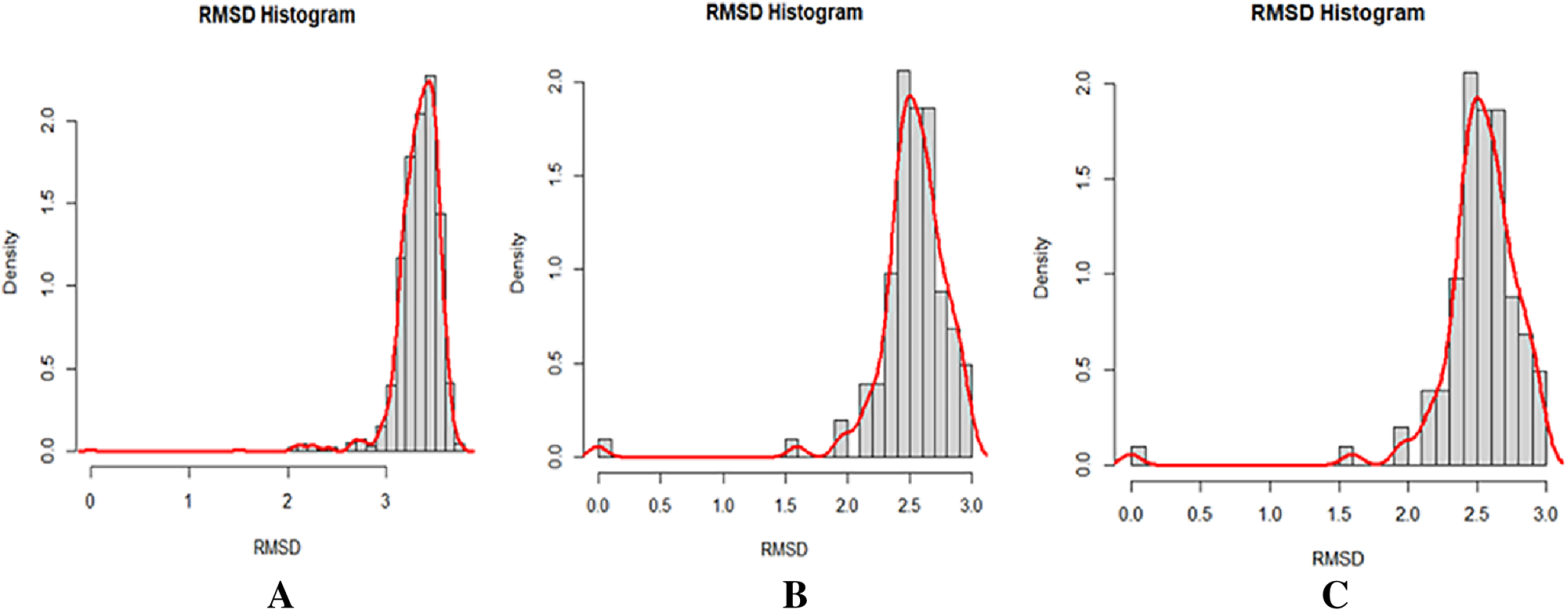
RMSD histograms show (**A**) Apigenin, (**B**) Apigenin-5-O-beta-d glucopyranoside, and (**C**) Apo protein.

It was determined that the average RMSD for the Apigenin-6L31 and Apigenin-5-O-beta-d-glucopyranoside -protein systems were 3.186 Å and 3.236 Å, respectively. The ligand-free protein, or apoprotein, has an RMSD value of 4.21 Å on average. For the apoprotein, we observed an abrupt increase in RMSD at the outset, followed by a sudden decrease 2.5 ns later. Following the trajectories, Apo form increased progressively for 20 ns, after which the value exceeded the RMSD of Apigenin, which may have occurred due to the higher occupancy of flexible loops in the C-terminal region and remained relatively stable until the end of the simulation. İn the case of the Apigenin complex system, a comparable deviation pattern was observed. The RMSD of apoprotein was greater during the 20 ns and 40 ns, and for the 40–50 ns, the value was very close to that of the apoprotein RMSD value. The RMSD of the Apigenin-6L31 system gradually increased and showed more fluctuations for 55 ns, after which the value attained its maximum at roughly 60 ns and declined to a lower value at 78.7 ns of the trajectory. After this time, the observed slight increased and became stable at 100 ns with negligible fluctuations. Whereas, the RMSD for the backbone atom of the Apigenin-5-O-beta-d-glucopyranoside complex concerning initial position quickly increased within the 20 ns run time and maintained a slightly non-significant fluctuation around 20 and 60 ns, where the following trajectories proceeded to drop slightly values till the end of the MD simulation. The average RMSD of the Apigenin-5-O-beta-D-glucopyranoside –6L31 is 0.82 greater than that of apoproteins; however, after 65 ns, the average RMSD value is less than that of apoproteins. In agreement with the above observation, it can be concluded that Apigenin complexes are very stable, as seen by the low RMSD value of Apigenin-5-O-beta-D-glucopyranoside − 6L31 after 60 ns and the constant RMSD value of Apigenin being very near to that of the apoprotein throughout the 100 ns MD simulation. Consequently, the RMSD of the Apigenin-6L31 and Apigenin-5-O-beta-D-glucopyranoside − 6L31 systems may suggest that they did not endure substantial conformational changes during the MD simulation. The RMSD histograms provided further evidence that the stability of the protein and ligand in the simulated system was seen and confirmed (Fig. 5A–C).

#### Root-mean-square fluctuation (RMSF)

**Figure 6:**
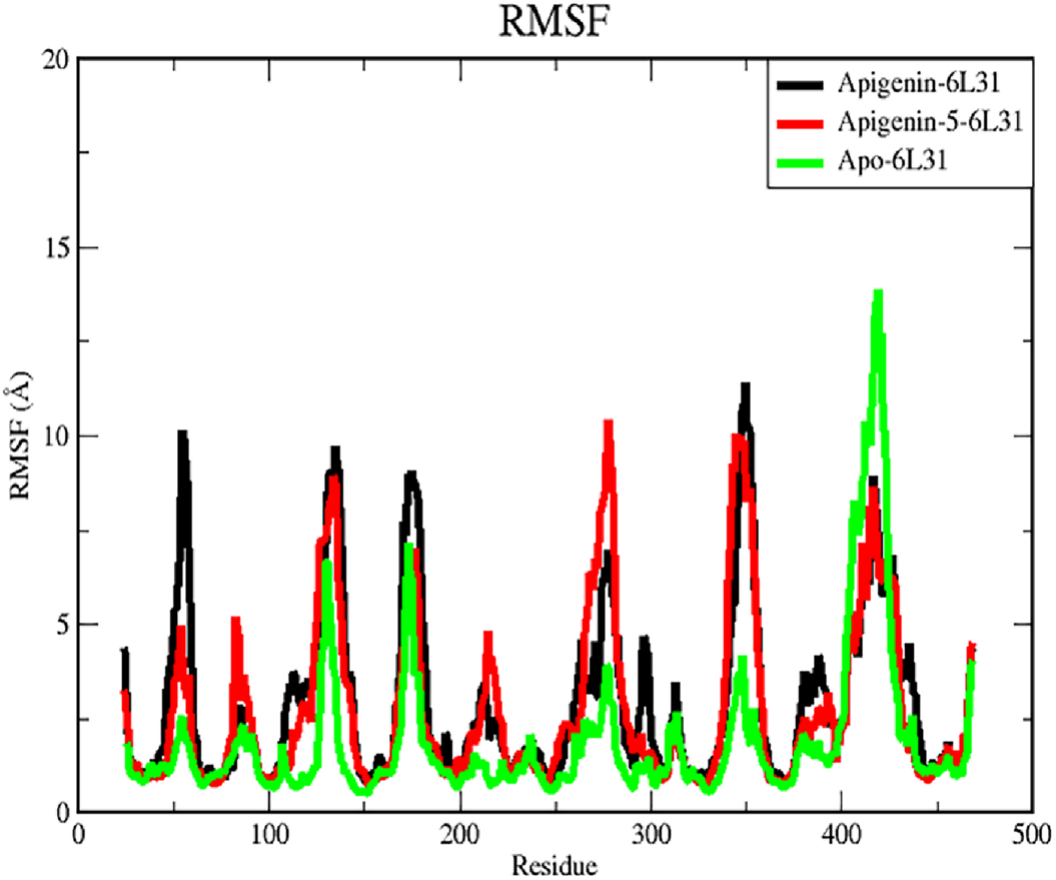
Displays the amino acid positional variation using RMSF analysis of Apo and ligand complexes in 100 ns molecular dynamics simulations.

The RMSF values are plotted to comprehend the residue-wise fluctuation between the apo and ligand complexes. The dynamic mobility in the loop sections is demonstrated by the RMSD deviation for the apo and complexes. For understanding the residues that participated in the causative factors for fluctuations, the RMSF plot is provided in Fig. 6. The RMSF value was utilized to identify the protein's hard and flexible regions. This validation criterion for structural variability in the ligand–protein complex highlights the importance of specific protein residues in these structural shifts. Using a timescale of 100 ns, the amino acid at each position is calculated for its deviation value.

For apoprotein, the amino acid position from 407 to 416 exhibits a deviation of up to 15 Å, whereas other amino acids exhibit deviations of 1 to 5 Å. The deviation that occurs in the 407–416th position and 1–5 A values may be the functional reason for the drift in apoprotein RMSD at 80th and 20 ns. In the process of comparing the values of the apo RMSF to those of the complex protein RMSF, it has been observed that Apigenin-6L31 demonstrates greater deviations than the other ligand complex. The amino acid positions from 55 to 57th, 131st to 137th, 173rd to 178th, 347th to 354th, and 417th to 418th have deviations ranging from 5 to 10. Apigenin-6L31’s RMSD deviates between the 55th and 60th nanoseconds as a result of these positional amino acid fluctuations. Similarly, another compound, Apigenin-5-O-beta-D glucopyranoside-6L31 also shows fluctuations in the same regions, only the amino acids between 277 and 280 exhibited higher RMSF value, which confirms the greater RMSD value between 40 and 60 ns in the RMSD plot. Higher RMSF values are indicative of a more flexible protein structure. The protein–ligand system produced RMSF values that were lower.

#### Radius of gyration (Rg) analysis

**Figure 7:**
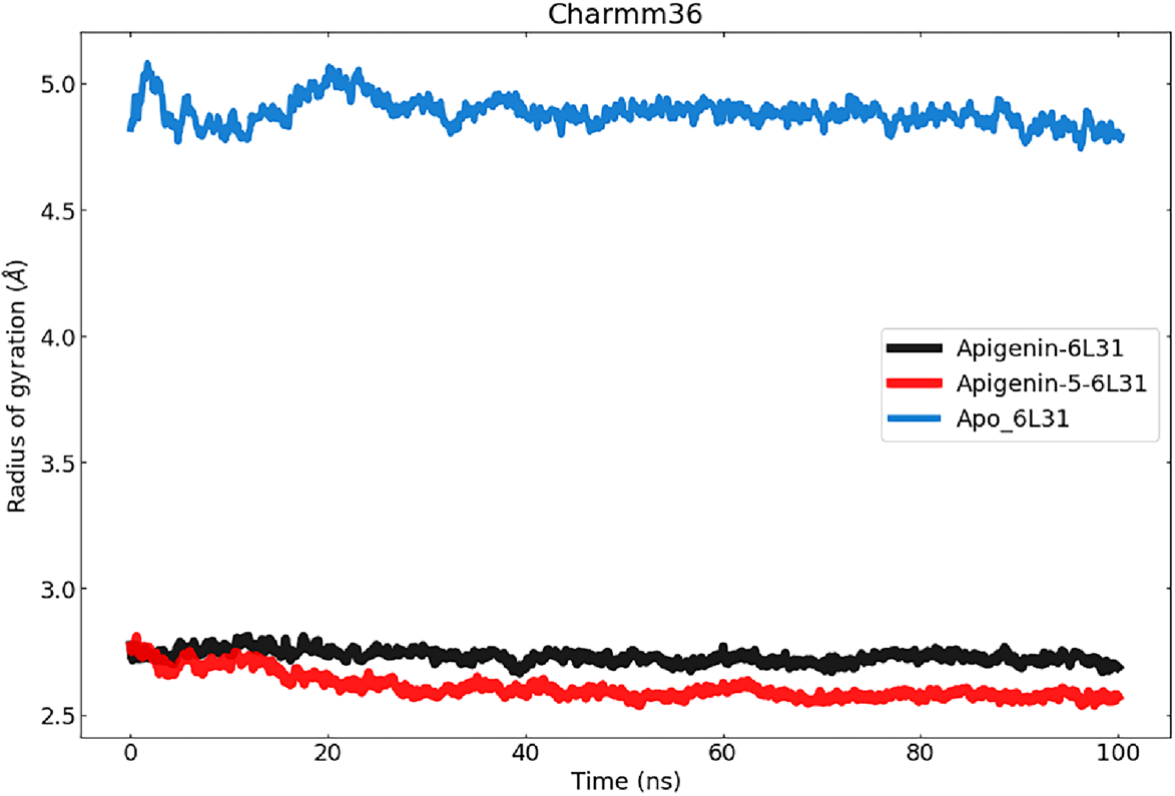
Represents the ROG values of the Apo-protein and protein–ligand complexes to the protein backbone for 100 ns. RoG of Apigenin-6L31, Apigenin-5-O-beta-D glucopyranoside -6L31, and Apo_6L31 are shown in black, red, and green respectively.

During the entirety of the simulation, the Rg parameter determines how compact a structure is. An increase in RoG values suggests a reduction in the compactness of the protein structure, indicating increased flexibility and decreased stability. The Rg-time fluctuations were observed to be nearly constant within the acceptable range, primarily maintained between 2.8 A and 2.6 A, indicating that the protein–ligand complexes undergo stable conformational changes. Compared to Apigenin-6L31 and apo_6L31, the radius of gyration was smallest for the Apigenin-5-O-beta-D glucopyranoside-6L31 complex (Fig. 7). According to the findings that were obtained, the Apigenin-5-O-beta-D glucopyranoside -6L31 complex was able to maintain a higher degree of stability during the simulation and bind successfully with the ligand. The trajectory of both proteins was used to produce a plot known as a solvent-accessible surface area (SASA), which was then used to research the proportion of each system's surface area that can be reached by the solvent (Fig. 7).

#### Solvent accessible surface area (SASA) analysis

**Figure 8:**
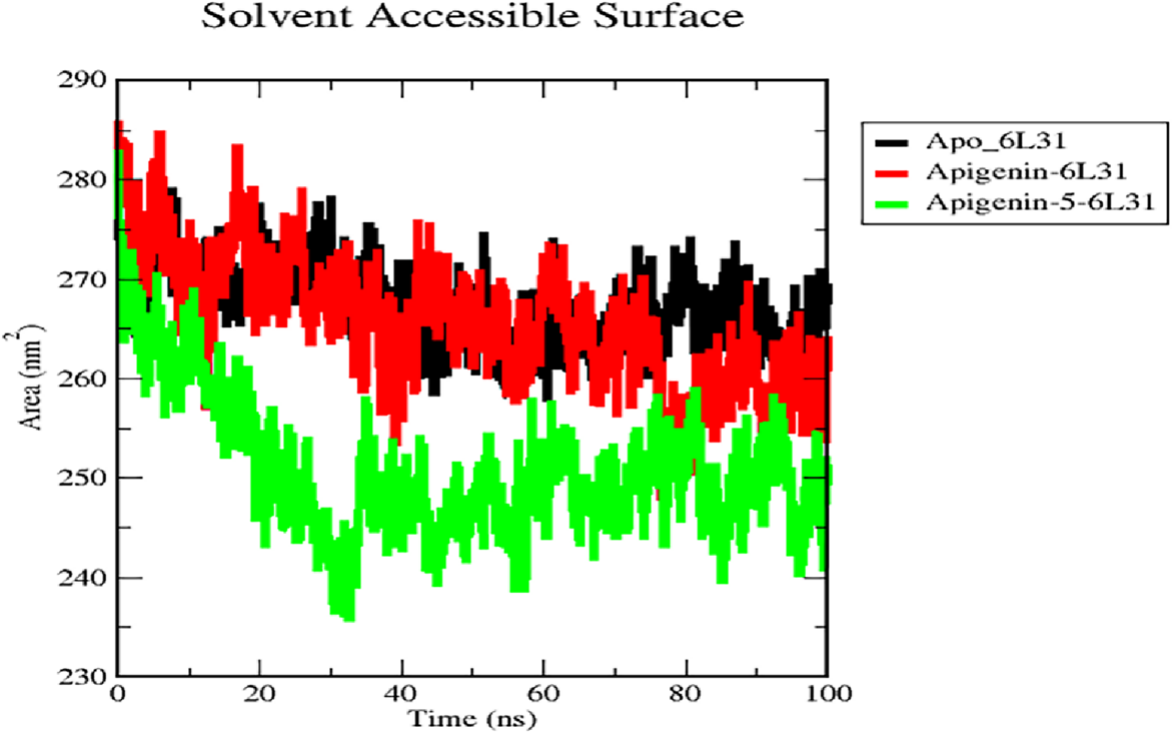
SASA analysis for the native structure of Apo_6L31 (black) and its complex with Apigenin (red) and Apigenin-5-O-beta-D glucopyranoside (green).

The information obtained from SASA will be useful for analyzing whether the ligand is kept inside the shallow binding pocket or whether it is expelled from the binding cavity. From Fig. 8, the SASA for the Apo_6L31, Apigenin-6L31 complex, and Apigenin-5-O-beta-d glucopyranoside − 6L31 complex, where the average SASA for the native protein was calculated to be 265.49, 261.037, and 255.149 nm2, was determined to be 265.49, 261.037, and 255.149 nm2, respectively. It has been shown that the Apigenin-5-6L31 complex displays a lower SASA in comparison to the Apo_6L31 and Apigenin-6L31 complexes; this indicates that the Apigenin-5-O-beta-D glucopyranoside -6L31 complex is responsible for inducing conformational alterations.

#### Hydrogen bond analysis

**Figure 9:**
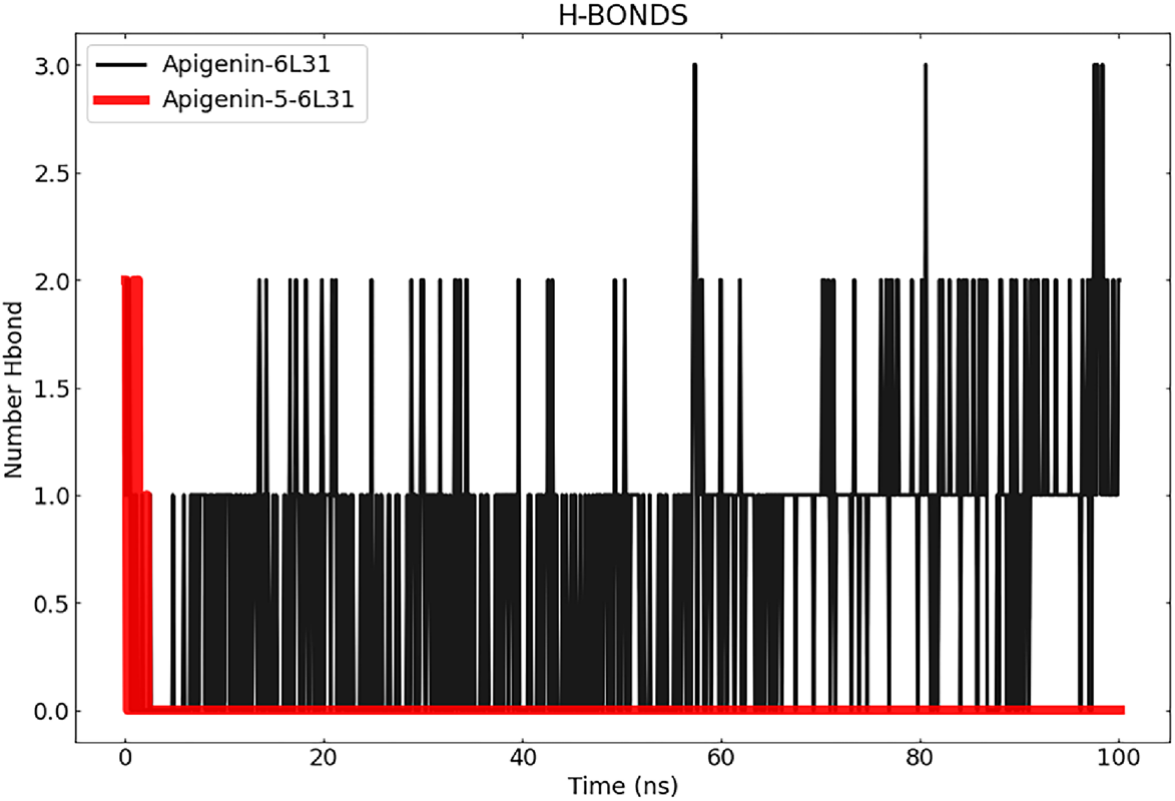
Represents the number of hydrogen bonds responsible for the stability of the complexes (Apigenin-6L31 and Apigenin-5-6L31) throughout the 100 ns.

Throughout the simulation, and in addition to the RMSD and SASA analyses, we also examined the stability of the hydrogen bonds (H-bonds) that are present in protein–ligand complexes. Understanding the connections between biomolecules necessitates a geometrical analysis of hydrogen bonding. Hydrogen bonds are an important interaction in maintaining the structural integrity of biomolecules. Also, During MD modeling, the creation of H-bonds is an essential component in maintaining the stability of the complexes, Throughout the entirety of the course of the MD simulation, it was discovered that the number of H-bonds that were present in the ligand-bound states was constantly changing, as shown in Fig. 9. The total number of H-bonds that were found between Apigenin and the protein during the MD simulation was 19, whereas the number of H-bonds that were found between Apigenin-5-O-beta-D glucopyranoside and the protein was a total of 8. It is evident from the graph that the Apigenin complex has more hydrogen bonds throughout the duration of the simulation, whereas the Apigenin-5-O-beta-d glucopyranoside -6L31 complex has fewer hydrogen bonds. In the instance of the Apigenin-5-O-beta-d glucopyranoside-6L31 mutant, it was discovered that there was a decrease in the number of hydrogen bonds when compared to Apigenin. Greater binding affinity is correlated with an increase in the number of hydrogen bonds formed and their duration. In addition, utilizing H-bond occupancy allowed for the identification of vital residues that were involved in the creation of H-bonds for ligand recognition. Using the VMD "Hydrogen bonds" tool, it was useful to explore the established ligand–protein hydrogen bond interactions and their relative frequencies^65^. The cut-off values for hydrogen bond (Donor H. Acceptor) distance and angle were assigned at 3.0 Å and 20°, respectively^66^.

Apigenin did not maintain all of the H-bonds that were detected in the docked complex, except Asn289; however, it did form additional contacts with Ile220, Asp215, Thr199, Cys225, Tyr147, Thr223, Thr224, Arg259, Gly264, Val267, and Glu265. Apigenin-5 maintained only its interactions with Asn289 and developed interactions with Tyr287, Phe201, Gly200, Trp165, and Tyr227 (Fig. 9), and Table 6 is displayed H-bond occupancy.

**Table 6:** H-bond occupancy.

Compound	Donor	Acceptor	Occupancy (%)
Apigenin	UNK0-Side-O4	ASP215-Side-OD2	23.95
	UNK0-Side-O4	ASP215-Side-OD1	17.56
	UNK0-Side-O3	ILE220-Main-O	8.98
	CYS225-Main-N	UNK0-Side-O3	5.89
	UNK0-Side-O4	ASP215-Main-O	5.79
	THR199-Side-OG1	UNK0-Side-O3	4.29
	UNK0-Side-O3	TYR147-Side-OH	0.90
	UNK0-Side-O4	ASN289-Main-O	0.20
Apiginenin-5	BGLC1-Side-O1	PHE201-Main-O	0.30
	BGLC1-Side-O6	ASN289-Main-O	0.30
	BGLC1-Side-O6	ASN289-Side-OD1	0.20
	BGLC1-Side-O2	TYR287-Main-O	0.20
	BGLC1-Side-O6	GLY200-Main-O	0.10

#### Contact frequency (CF) analysis

**Figure 10:**
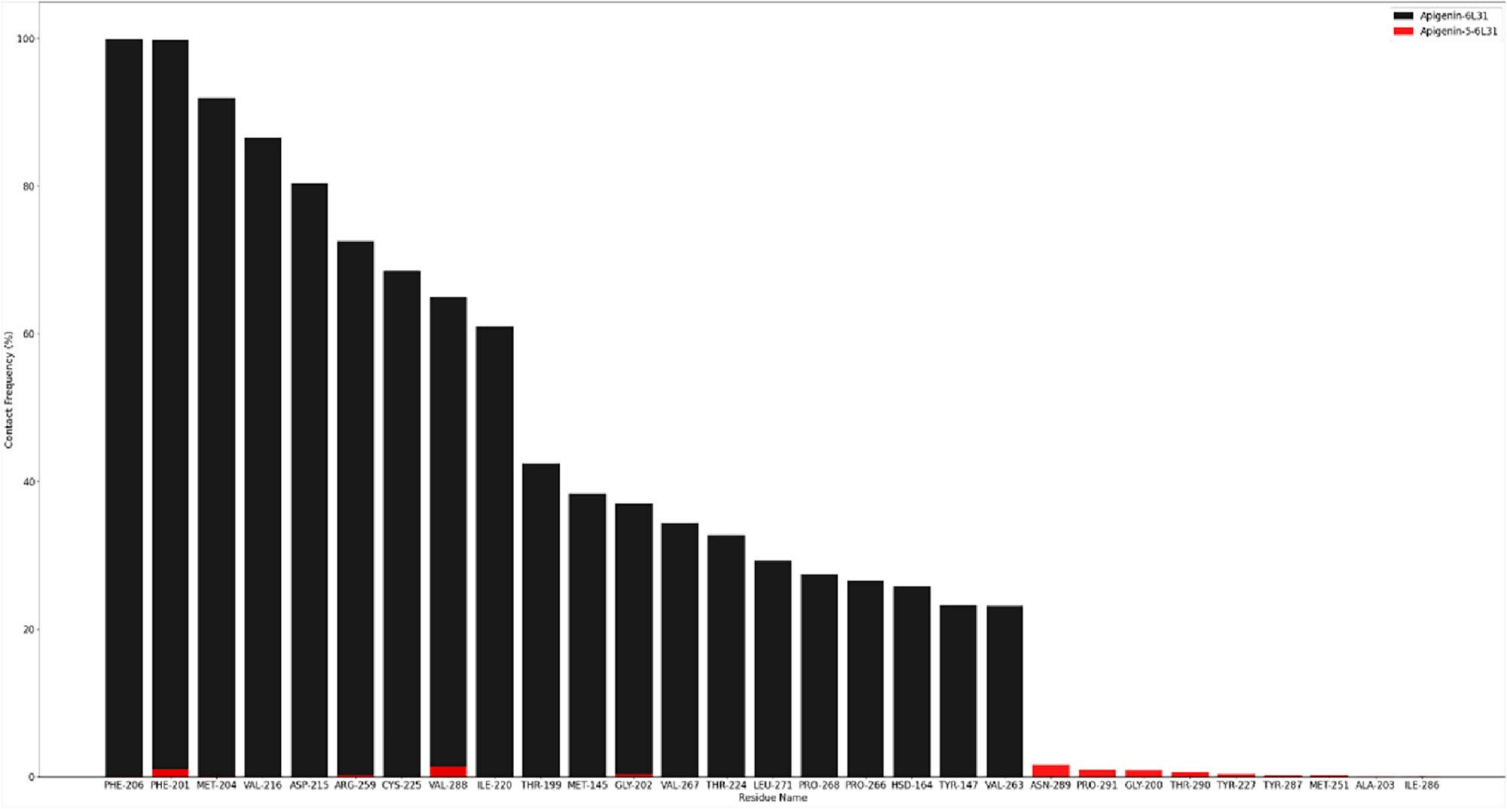
Contact frequency analysis during the MD simulation. A contact frequency plot of 6L31 residues interacting with Apigenin (black) and Apigenin-5-O-beta-d glucopyranoside antigen (red).

To, Fig. 10 depicts the results of a contact frequency (CF) analysis performed with the contact Freq module on VMD and a cut-off of 4 to further evaluate the binding between 6L31 and the ligands. Phe206, Phe201, Met204, Val216, Asp215, Arg259, Cys225, Val288, Ile220, Thr199, Met145, Gly202, Val267, Thr224, Leu271, Pro268 and Pro266, Hsd164, Tyr147, and Val263 had the highest CF during the simulation. Apigenin exhibited a higher overall contact frequency with these residues than Apigenin-5. Moreover, the CFs of Phe201, Val288, and Gly202 with Apigenin and Apigenin-5 were quite distant. The hydrogen bonds formed between protein and apigenin ligand were not the same as those formed between apigenin-5 and protein, except asn289. In addition, van der Waals interactions were observed between Phe206, Phe201, Val216, Ile220, and Val288 with both ligands, all of which were identified in the presented high CF. Based on the H-bond analysis, we can conclude that Apigenin complex binds to protein active sites more efficiently and tightly than Apigenin-5-6L31. Additionally, the presence of hydrogen bonds between 6L31 and Apigenin derivatives has helped to strengthen the binding, which has contributed to the simulation's success in maintaining its stability (Fig. 10).

### Principal component analysis (PCA)

**Figure 11:**
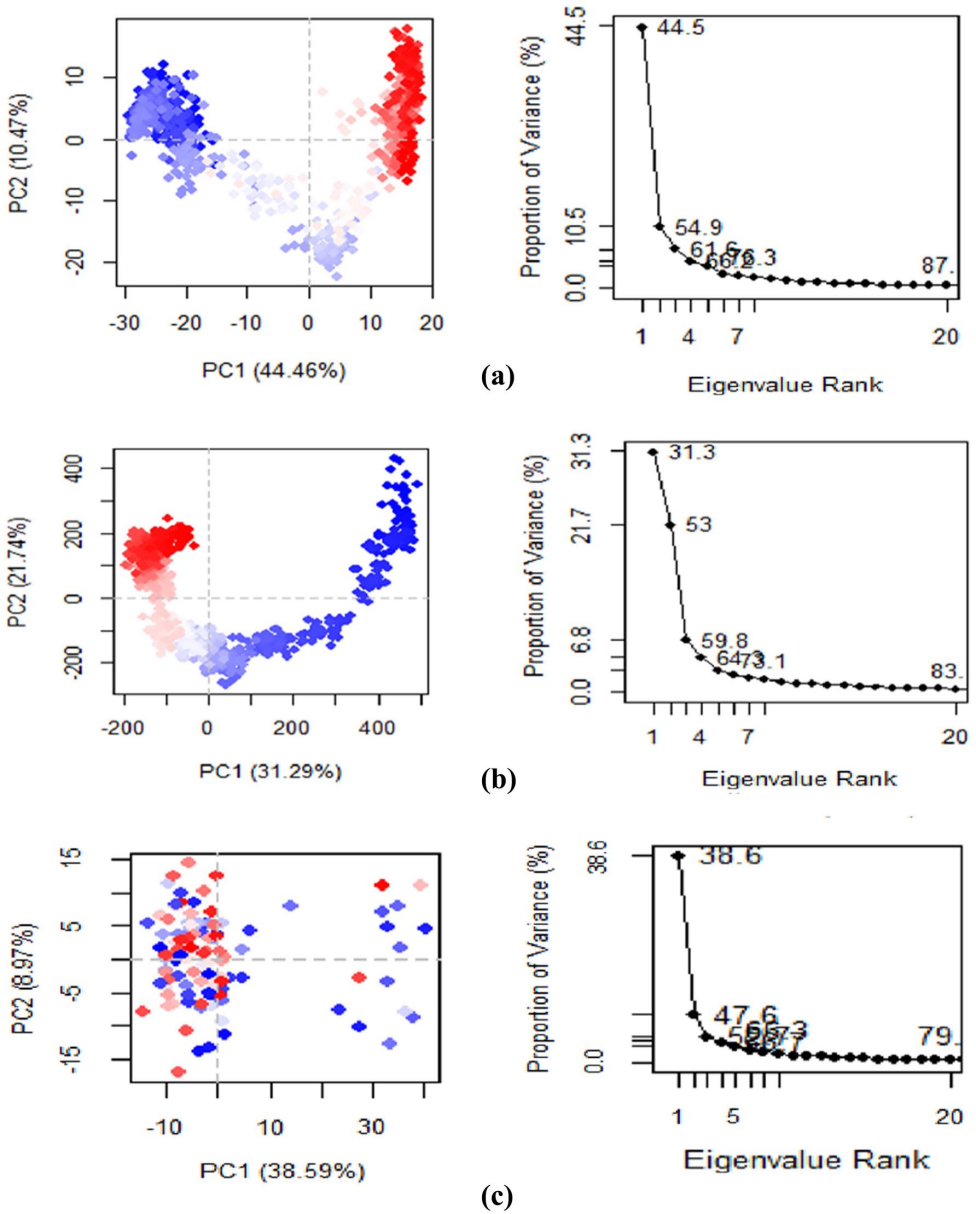
Principal component analysis of (**a**) Apigenin, (**b**) Apigenin-5, and (**c**) Apo protein. Each point represents the protein’s conformation on the X and Y axes. The chromatic distribution of blue and red dots was utilized to depict the extent of conformational alterations in the simulation. The color gradient ranging from blue to white to red was indicative of the duration of the simulation. The color blue designates the initial timestep, while the color white represents the intermediate timestep, and the color red signifies the final timestep.

Principal Component Analysis (PCA) is a useful technique for extracting crucial information from Molecular Dynamics (MD) trajectories by modifying global slow motions from local fast motions. It was utilized to model the significant dynamics of both the complex systems and the apo protein to investigate the nature of the interaction between the statistically significant conformations that were found along the trajectory. The complex's essential variations were captured by arranging the principal components as eigenvectors according to their variance. Eigenvalue rank plots display the fraction of total variation explained by each component. The PCA plots for the c-alpha backbone of the protein in the complex of Apigenin, Apigenin-5-O-beta-D glucopyranoside, and 6L31-apo are shown in Fig. 11. Predicting the significant motions in the trajectory is useful. PCA was performed using RStudio and Bio3d^67^. Dynamic simulations are essential to biological function, and PCA can isolate the most variable of these motions to investigate the conformational change of the systems, the PCA scatter plots of the 6L31 apo, Apigenin, and Apigenin-5-O-beta-D glucopyranoside systems were generated by projecting the simulated trajectories of the protein systems into the two-dimensional subspace spanned by the first three eigenvectors (PC1, PC2, and PC3). This allowed the conformational change of the systems to be investigated. Figure X displays the principal component analyses (PCA) that reveal that the 6L31 apo, Apigenin, and Apigenin-5-O-beta-d glucopyranoside systems each contributed 44.6%, 31.29%, and 38.89 (14.29)% of the total variations, respectively. The Apigenin complex was found to have the highest PC1 value (44.6%), which suggests that the complex has been subjected to a greater number of conformational changes. In contrast, the Apigenin-5 complex exhibits less PC1 (31.29%), indicating that it has undergone a smaller conformational change. Moreover, the PC1 of the Apo structure was 38.9%, which is greater than the Apigenin-5-O-beta-D glucopyranoside complex, indicating that the binding of Apigenin-5-O-beta-d glucopyranoside stabilizes the Apo's conformational changes. Figure 11(a, b and c) shows the conformational state of the three systems in the subspace as depicted by the principal component analysis scatter diagram, with the red dot representing the stable conformation, the blue dot representing the unstable conformation, and the white dots representing the intermediate state between the three conformations.

### Dynamic cross-correlation matrix (DCCM) analysis

**Figure 12:**
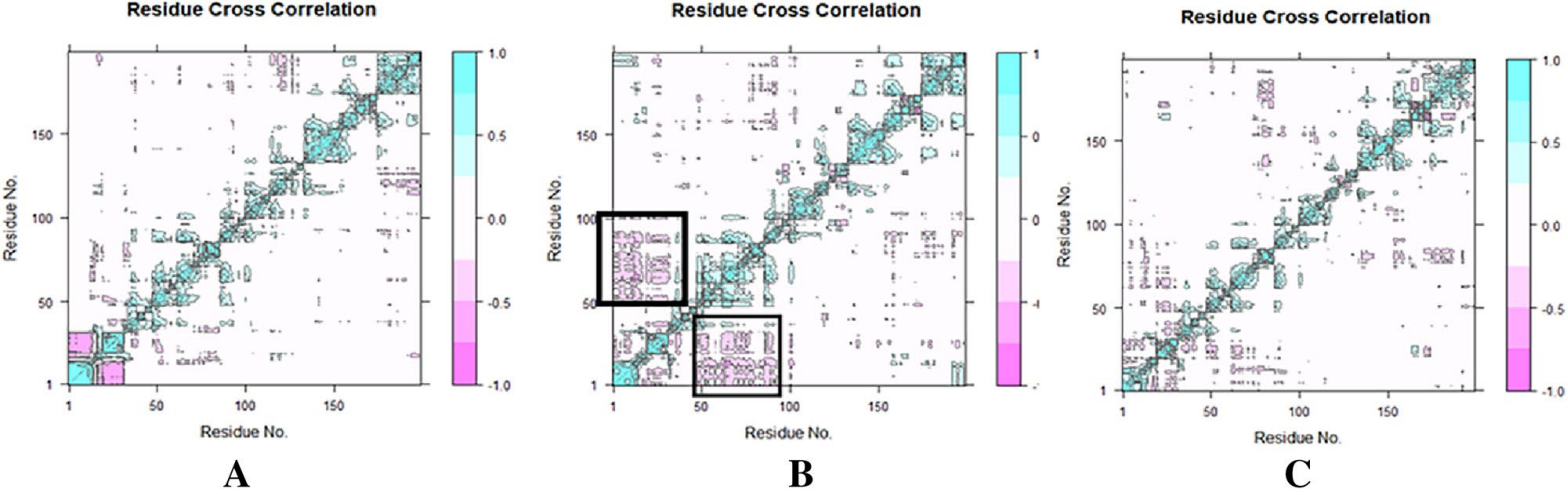
Ca-residue cross-correlation profiles for (**A**) Apigenin, (**B**) Apigenin-5-O-beta-D glucopyranoside proteins, and (**C**) Apo protein [L1 protein of human papillomavirus (PDB ID 6L31)].

To investigate the effect of Apigenin derivatives on the conformational motions of the 6L31 protein, DCCM analyses were undertaken on all C atoms in the 6L31 apo, the Apigenin complex, and the Apigenin-5-O-beta-D glucopyranoside complex system using 100 ns simulated trajectories (Fig. 12a, b and c). The DCCM exhibited a comprehensive correlation, encompassing a range of values from − 1.0 to 1.0, with the former indicating a dark purple hue and the latter indicating a dark blue hue. It was determined that different shades of color correspond to varying degrees of correlation between residues, with the deeper the color indicating a larger degree of association. The observed correlation coefficient, ranging from − 1 to 1, indicated that residues exhibited either a positive or negative relationship in their movements. A positive correlation indicated that residues moved in the same direction, while a negative correlation indicated that residues moved in opposite directions. Upon analyzing the DCCM diagrams of the three systems, it was observed that the correlated movements exhibited by each system were notably distinct. In contrast to the Apigenin complex system, the collective movements that exhibit positive correlation in the entire Apigenin-5-O-beta-D glucopyranoside complex remained relatively stable, while the movements that display negative correlation experienced a notable increase. The correlated movements of the Apigenin-5-O-beta-d glucopyranoside complex exhibit significant changes upon ligand binding, particularly in marked areas denoted by black dashed boxes.

### The binding free energy estimation

**Figure 13:**
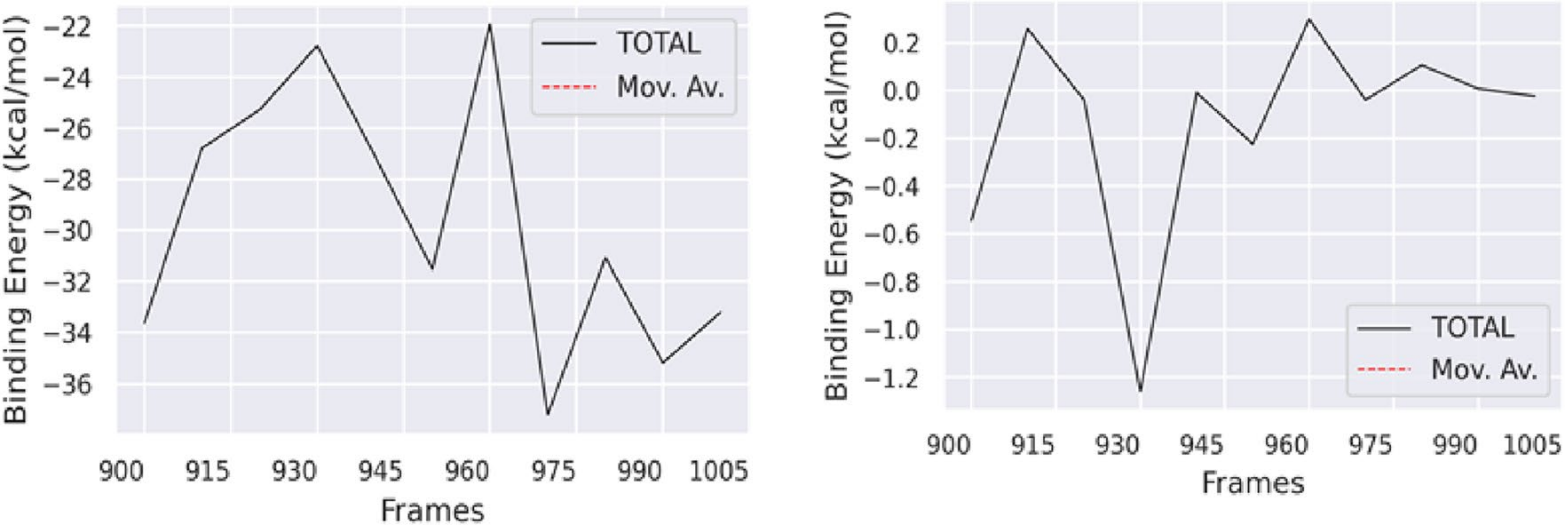
Binding free energy plot.

The MM/PBSA approach is a noteworthy technique utilized for the computation of the binding free energy of protein–ligand complexes. The MM-PBSA method was employed to determine the binding free energy of the final 20 compounds based on the molecular dynamics (MD) trajectories. The value of ΔG is determined by the collective impact of diverse protein–ligand interactions, including but not limited to van der Waals energy (ΔEvdW), electrostatic energy (ΔEele), and EPB (electrostatic contribution to solvation-free energy by Poisson-Boltzmann) energy (Fig. 13).

The binding energies of the Apigenin complex is − 29.61 kj/mol found in whereas for Apigenin-5-O-beta-d glucopyranoside complex is − 0.13 kJ/mol. The Apigenin, exhibited ∆VDW (− 35.52 kcal/mol), ∆EEL (− 23.39 kcal/mol), and ∆EGB (32.49 kcal/mol), while compound Apigenin-5 11 reflected ∆VDW (− 0.40 kcal/mol), ∆EEL (0.09 kcal/mol) and ∆EGB (0.29 kcal/mol) energies of completely different. The MM-PBSA analysis yielded findings indicating that Apigenin exhibited robust binding energy and greater stability. The validation of the outcomes of the molecular docking and MD simulations were carried out through the binding free energy calculation.

### Dynamic behavior and confirmational change of protein–ligand complex

**Figure 14:**
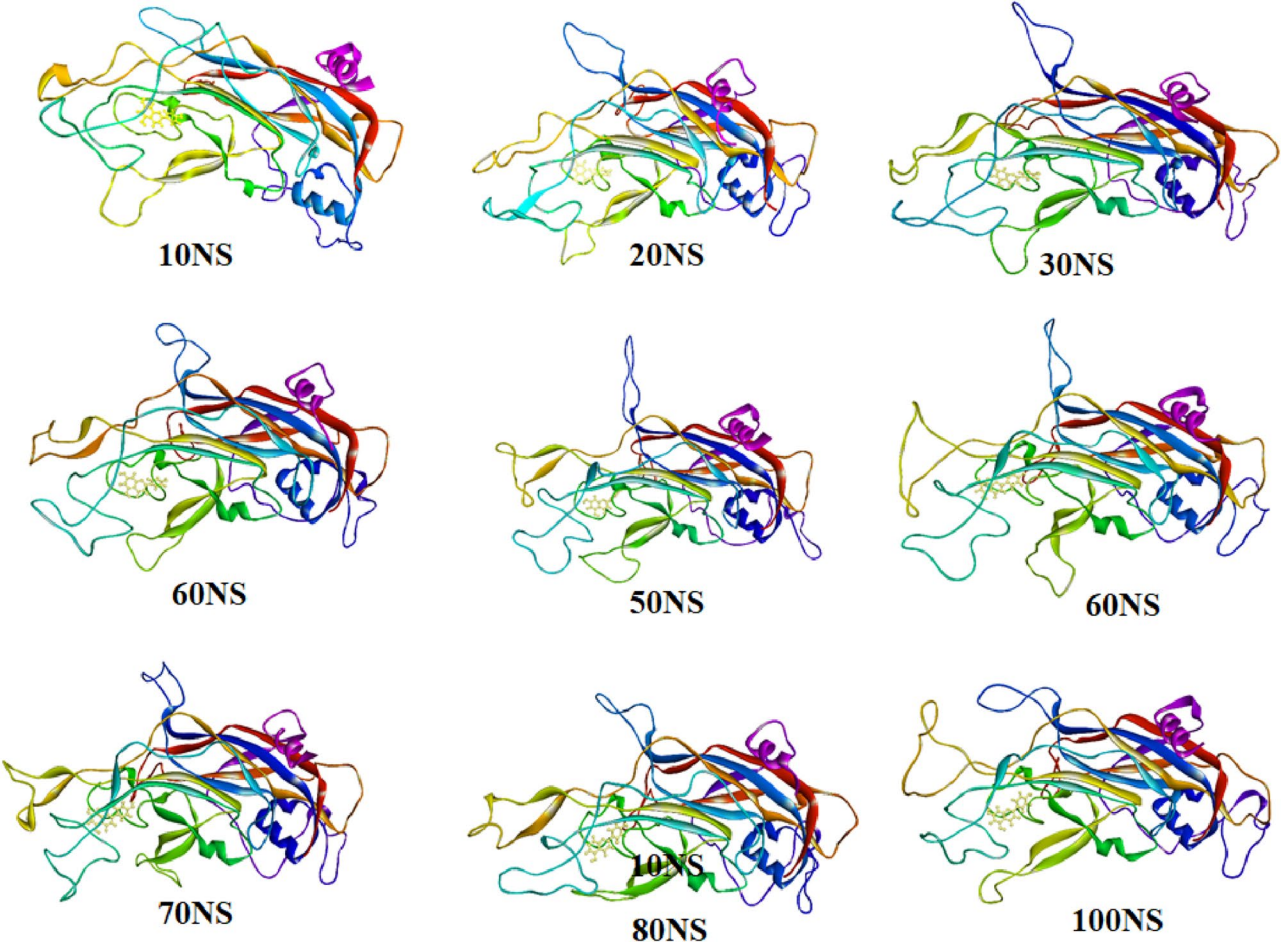
Time frame analysis at every 10 ns of Apigenin complex with L1 protein of human papillomavirus (PDB ID 6L31).

To understand the dynamic structural evolution of the L1 protein of human papillomavirus (PDB ID 6L31)-ligand throughout the 100 ns simulation time frame, nine snapshots at every 10 ns have been taken. Additionally, it has been shown that the ligands remain entirely attached to the inhibitory site without undergoing any structural alteration, suggesting that they are quite stable (Fig. 14).

### Frontier molecular orbital analysis (FMO)

**Figure 15:**
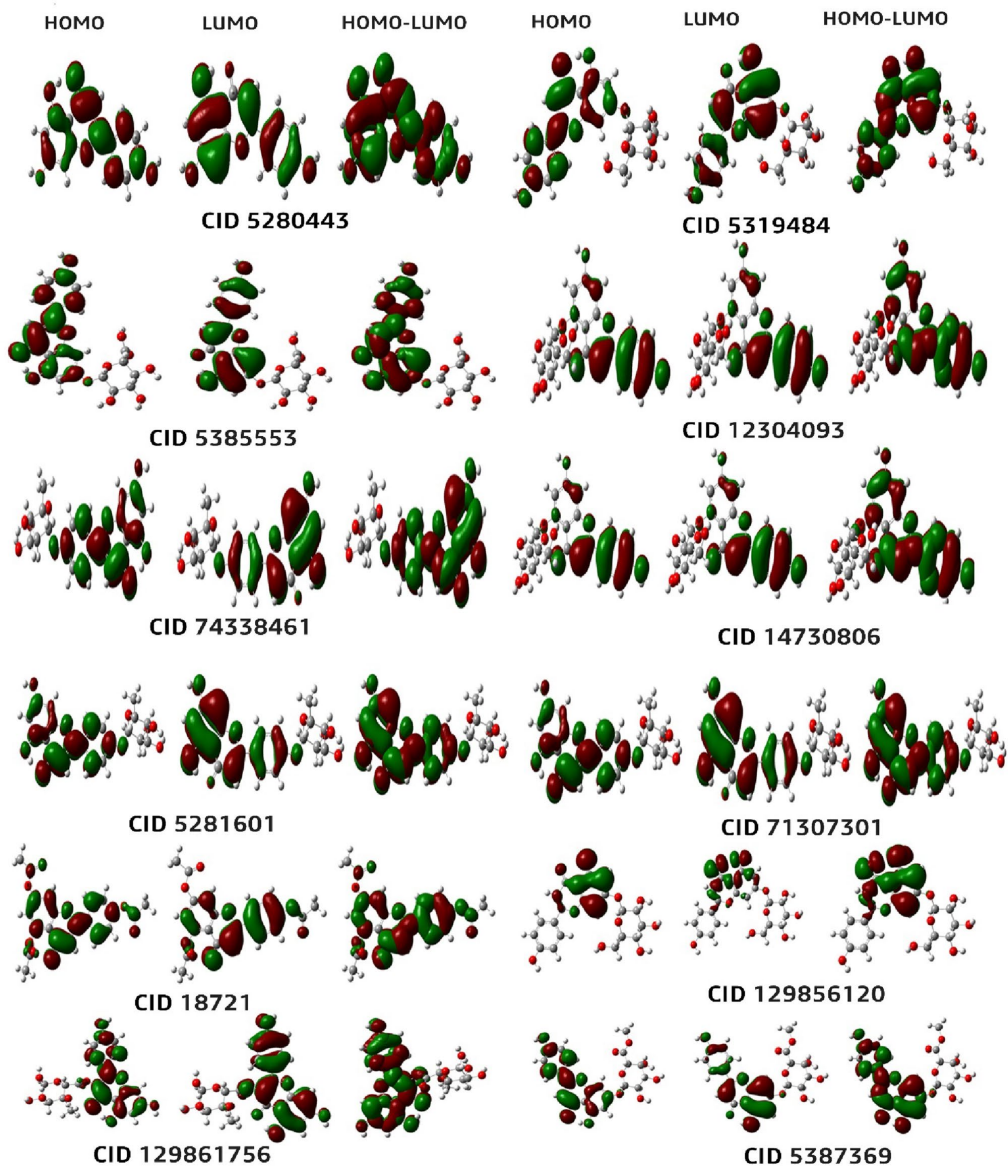
Diagram of the frontier molecular orbital.

The present investigation employed the Density Functional Theory (DFT) methodology based on quantum mechanics to compute the HOMO and LUMO energy of twelve compounds. The outcome of this analysis is depicted in Fig. 8. The frontier orbitals, specifically the highest occupied molecular orbital (HOMO) and the lowest occupied molecular orbital (LUMO) can be utilized to characterize the reactivity of chemical species^68^. The highest occupied molecular orbital (HOMO) and lowest unoccupied molecular orbital (LUMO) are utilized to characterize the electron-donating and accepting properties of chemical compounds. An additional parameter that warrants consideration is the energy gap, denoting the disparity between the highest occupied molecular orbital (HOMO) and the lowest unoccupied molecular orbital (LUMO) energies. This differential is indicative of intramolecular charge transfer and kinetic stability. Compounds possessing a significant energy gap exhibit reduced chemical reactivity and heightened kinetic stability. In contrast, individuals possessing a narrow energy gap exhibit heightened reactivity and diminished kinetic stability. In this study, the HOMO and LUMO energies of twelve compounds were calculated using the quantum mechanical Density Functional Theory (DFT) method, and the result is shown in Fig. 15.

### Chemical reactivity and molecular properties analysis

According to the findings, the compound Apigenin-7-O-Methyl Glucuronate exhibited the smallest energy gap (ΔE) in comparison to the other compounds. This indicates a heightened level of chemical reactivity and significant intramolecular charge transfer from an electron donor (HOMO) to an electron acceptor (LUMO) group. The present study examined two compounds, Apigenin and apigenin-5, and found that Apigenin exhibits a comparatively slightly lower energy gap than Apigenin 5-O-Beta-D-Glucopyranoside. A molecule with a larger HOMO–LUMO energy gap indicates high chemical inertness and instability^69^. The principal factor behind this phenomenon is the obstruction of the electronic transition, which is caused by a significant energy differential between the ground state and the excited state. Typically, a molecule exhibiting a small HOMO–LUMO gap indicates high stability.

Based on the findings presented in Table 7, it can be observed that the HOMO–LUMO gaps of all the chemicals under investigation fall within the range of 3.960 eV to 4.079 eV. Furthermore, the data indicates that the order of the energy gap follows a descending pattern as follows: 10 > 9 > 6 > 2 > 7 > 1 > 8 > 5 > 3 > 4 > 12 > 11. The value of the softness is displayed in Table 7. It is essential to keep in mind that the disintegration time required for an element will be shorter and that it will deteriorate at a faster pace than that of other elements if its softness level is larger than a tiny value. On the other hand, the property of hardness is a fundamental characteristic of a substance, and its quantification serves as an indicator of its durability. Typically, compounds with higher hardness values exhibit greater resistance to alterations in electron configuration at the molecular level. Our reported molecules have shown 11 > 12 > 04 > 03 > 08 > 05 > 01 > 07 > 02 > 06 > 09 > 10, which means compounds 11, 12, and 04 will more rapidly disintegrate compared to the other molecules. Again, the hardness is always opposite to softness, and the hardness values are reported as 10 > 09 > 06 > 02 > 07 > 01 > 05 > 08 > 03 > 04 > 12 > 11 in our studies, which indicates that the compounds 11, 12 and 04 are lower hardness and ultimately disintegrate quickly.

**Table 7:** Chemical reactivity and molecular properties data.

No	Name	A = –LUMO eV	I =–HOMO eV	Energy = I-A eV	Hardness	Softness
01	5280443	− 2.06501	− 6.1195	4.05531	2.02724	0.4933
02	5319484	− 2.17800	− 6.2578	4.07980	2.0399	0.4902
03	5385553	− 1.91758	− 5.9680	4.05041	2.02521	0.4938
04	12304093	− 2.06833	− 6.0988	4.03055	2.01523	0.4962
05	74338461	− 2.10969	− 6.1612	4.05150	2.02575	0.4936
06	14730806	− 1.96058	− 6.2134	4.25280	2.12641	0.4703
07	5281601	− 1.97554	− 6.0455	4.07000	2.03498	0.4914
08	71307301	− 2.10997	− 6.1612	4.05123	2.025615	0.4937
09	18721	− 2.17636	− 6.4436	4.26892	2.13362	0.4687
10	129856120	− 1.75186	− 6.1010	4.34919	2.17457	0.4599
11	129861756	− 2.24521	− 6.2052	3.96007	1.979995	0.5051
12	5387369	− 1.90425	− 5.9247	4.02048	2.010225	0.4975

## Conclusion

The effectiveness of Apigenin derivatives has been utilized in this current investigation as the proposed compounds for the novel treatments for HPV-associated cervical cancer and the DNA polymerase theta since there is no targeted therapy for them. This research gap encourages our research team to develop an urgent search for the potential molecules against them with novel modes of action. So, this in silico study has been performed to screen potential drug candidates from a series of Apigenin derivatives with significant pharmacological properties. This current investigation also includes the pharmacokinetic properties, drug-likeness, ADMET profiles, molecular docking, molecular dynamic simulation, PCA, DCCM DFT, and QSAR. The molecular dynamics simulation, and molecular docking, methods were employed to prove the binding affinities against targeted receptors and the stability of the compounds. The results showed that all the derivatives of the Apigenin molecule are drug-like, and promising hydrogen bonding was reported, exhibiting remarkably inhibitory capability for each of the targeted receptors and favorable binding energies. The favorable rate-determining binding affinities across targeted protein ranges are − 7.1 kcal/mol to − 9.3 kcal/mol. It is also noted that Apigenin 5-O-Beta-d-Glucopyranoside was reported maximum affinities (− 9.3 kcal/mol) against the L1 protein of human papillomavirus (PDB ID 6L31). Finally, our investigation found that the Apigenin derivatives should be suggested as a novel compound against HPV-associated cervical cancer and the DNA polymerase theta. It is kept in mind that these advanced computational studies are provided the potential activity theoretically. Further wet lab experiments should be conducted to validate this effect in vitro, in vivo, pre-clinical, and clinical trials.

### Limitations of the study

It is a theoretical investigation; to validate this investigation, and develop newer and safer drugs from the synthetic sources, these derivatives must be carried out from computational to (in vitro and in vivo), preclinical and clinical trials, to find out their practical value.

## Data Availability

All data generated or analyzed during this study are included in this published article.

## Abbreviations

ADMET: Absorption, distribution, metabolism, excretion and toxicity
ADP-ribose: Adenosine diphosphate ribose
ARG: Arginine
ASN: Asparagine
ASP: Aspartic acid
ATPase: Adenosine diphosphatase
BBB: Blood brain barrier
Bp: Base pairs
BRCA1: Breast cancer 1
BRCA2: Breast cancer 2
CHARMM36: Chemistry at harvard macromolecular mechanics
CYS: Cysteine
DCCM: Dynamic cross-correlation matrix Bio3D
DFT: Density functional theory
*DNA*: Deoxyribonucleic acid
ELISA: Enzyme linked immunosorbent assay
EPB: Export promotion bureau
FMO: Frontier molecular orbitals
GLN: Glutamine
GLU: Glutamic acid
GLY: Glycine
GNU: General public license
GROMACS-2019: GROningen machine for chemical simulations-2019
H-bond: Hydrogen bond
HIS: Histidine
HOMO: Highest occupied molecular orbital
HPV: Human papillomavirus
HR: Homologous recombination
HRR: Homologous recombinational repair
ILE: Isoleucine
kcal/mol: Kilo calorie mole
LCR: Long control region
LEU: Leucine
LUMO: Lowest occupied molecular orbital
LYS: Lysine
MDs: Molecular dynamics simulation
MET: Methionine
MLR: Multiple linear regression
MMEJ: Microhomology mediated end joining
MM-PBSA: Molecular mechanics Poisson-Boltzmann surface area
OCT2: Organic cation transporter 2
ORFs: Open reading frames
P97: Protein 97
PARPi: Poly adenosine diphosphate ribose polymerase
PCA: Principal component analysis
PCR: Polymerase chain reaction
PHE: Phenylalanine
pkCSM: Predicting small molecule pharmacokinetic and toxicity properties using graph-based signatures
PME: Particle mesh Ewald
Pol θ: DNA polymerase theta
PRO: Proline
QSAR: Quantitative structure activity relationship
RCSB: Research collaboratory for structural bioinformatics
RMSD: Root Mean square deviation
RMSF: Root Mean square fluctuation
RoG profiles: Radius of gyration
RPA: Replication protein A
SASA: Solvent accessible surface area
SER: Serine
SMILES: Simplified molecular input line entry system
THR: Threonine
TIP3P: Transferable intermolecular potential with 3 point
TRP: Tryptophan
VAL: Valine
VMD: Visual molecular dynamics

## References

[1] Poniewierza P, Panek G (2022). Cervical cancer prophylaxis—State-of-the-art and perspectives. Healthcare.

[2] Rahib L, Wehner MR, Matrisian LM, Nead KT (2021). Estimated projection of US cancer incidence and death to 2040. JAMA Netw. Open.

[3] Chan CK, Aimagambetova G, Ukybassova T, Kongrtay K, Azizan A (2019). Human papillomavirus infection and cervical cancer: Epidemiology, screening, and vaccination—Review of current perspectives. J. Oncol..

[4] Jalil, A. A. T. Epidemiology of Cervical cancer and high risk of human papilloma virus in patient. *ББК 28.6 З,* 85, 7.

[5] Kanda T, Kukimoto I (2006). Human papillomavirus and cervical cancer. Uirusu.

[6] Gravitt PE, Burk RD, Lorincz A, Herrero R, Hildesheim A, Sherman ME (2003). A comparison between real-time polymerase chain reaction and hybrid capture 2 for human papillomavirus DNA quantitation. Cancer Epidemiol. Biomark. Prev..

[7] Kelesidis T, Aish L, Steller MA, Aish IS, Shen J, Foukas P (2011). Human papillomavirus (HPV) detection using in situ hybridization in histologic samples: Correlations with cytologic changes and polymerase chain reaction HPV detection. Am. J. Clin. Pathol..

[8] Watts DH, Koutsky LA, Holmes KK, Goldman D, Kuypers J, Kiviat NB (1998). Low risk of perinatal transmission of human papillomavirus: Results from a prospective cohort study. Am. J. Obstet. Gynecol..

[9] Einstein MH, Goldberg GL (2002). Human papillomavirus and cervical neoplasia. Cancer Investig..

[10] Thompson LH, Schild D (2001). Homologous recombinational repair of DNA ensures mammalian chromosome stability. Mutat. Res. Fundam. Mol. Mech. Mutagenes..

[11] Hoppe MM, Sundar R, Tan DSP, Jeyasekharan AD (2018). Biomarkers for homologous recombination deficiency in cancer. J. Natl. Cancer Inst..

[12] Lord CJ, Ashworth AJS (2017). PARP inhibitors: Synthetic lethality in the clinic. Science.

[13] Bryant HE, Schultz N, Thomas HD, Parker KM, Flower D, Lopez E (2005). Specific killing of BRCA2-deficient tumours with inhibitors of poly (ADP-ribose) polymerase. Nature.

[14] Farmer H, McCabe N, Lord CJ, Tutt AN, Johnson DA, Richardson TB (2005). Targeting the DNA repair defect in BRCA mutant cells as a therapeutic strategy. Nature.

[15] Ledermann J, Harter P, Gourley C, Friedlander M, Vergote I, Rustin G (2012). Olaparib maintenance therapy in platinum-sensitive relapsed ovarian cancer. N. Engl. J. Med..

[16] Coleman RL, Oza AM, Lorusso D, Aghajanian C, Oaknin A, Dean A (2017). Rucaparib maintenance treatment for recurrent ovarian carcinoma after response to platinum therapy (ARIEL3): A randomised, double-blind, placebo-controlled, phase 3 trial. Lancet.

[17] Mirza MR, Monk BJ, Herrstedt J, Oza AM, Mahner S, Redondo A (2016). Niraparib maintenance therapy in platinum-sensitive, recurrent ovarian cancer. N. Engl. J. Med..

[18] Litton JK, Rugo HS, Ettl J, Hurvitz SA, Gonçalves A, Lee K-H (2018). Talazoparib in patients with advanced breast cancer and a germline BRCA mutation. N. Engl. J. Med..

[19] Zhou J, Gelot C, Pantelidou C, Li A, Yücel H, Davis RE (2021). A first-in-class polymerase theta inhibitor selectively targets homologous-recombination-deficient tumors. Nat. Cancer.

[20] Carvajal-Garcia J, Crown KN, Ramsden DA, Sekelsky J (2021). DNA polymerase theta suppresses mitotic crossing over. PLoS Genet..

[21] Wood RD, Doublié S (2016). DNA polymerase θ (POLQ), double-strand break repair, and cancer. DNA Repair.

[22] Seki M, Marini F, Wood RD (2003). POLQ (Pol θ), a DNA polymerase and DNA-dependent ATPase in human cells. Nucleic Acids Res..

[23] Ceccaldi R, Liu JC, Amunugama R, Hajdu I, Primack B, Petalcorin MI (2015). Homologous-recombination-deficient tumours are dependent on Polθ-mediated repair. Nature.

[24] Mateos-Gomez PA, Gong F, Nair N, Miller KM, Lazzerini-Denchi E, Sfeir AJN (2015). Mammalian polymerase θ promotes alternative NHEJ and suppresses recombination. Nature.

[25] Chan SH, Yu AM, McVey M (2010). Dual roles for DNA polymerase theta in alternative end-joining repair of double-strand breaks in Drosophila. PLoS Genet..

[26] Higgins GS, Boulton SJ (2018). Beyond PARP—POLθ as an anticancer target. Science.

[27] Rivera A, Tyring SK (2004). Therapy of cutaneous human papillomavirus infections. Dermatol. Ther..

[28] Kumer A, Chakma U, Rana MM, Chandro A, Akash S, Elseehy MM (2022). Investigation of the new inhibitors by sulfadiazine and modified derivatives of α-D-glucopyranoside for white spot syndrome virus disease of shrimp by in silico: Quantum calculations, molecular docking, ADMET and molecular dynamics study. Molecules.

[29] Rahman MM, Karim MR, Ahsan MQ, Khalipha ABR, Chowdhury MR, Saifuzzaman M (2012). Use of computer in drug design and drug discovery: A review. Int. J. Pharm. Life Sci..

[30] Lowy DR, Schiller JT (2006). Prophylactic human papillomavirus vaccines. J. Clin. Investig..

[31] Ferraro CTL, Canedo NHS, de Oliveira SP, da Glória da Costa Carvalho M, Dias EP (2011). HPV oral infection and proliferative epithelial associated lesions. J. Bras. Patol. Med. Lab..

[32] Cai Q, Lv L, Shao Q, Li X, Dian A (2013). Human papillomavirus early proteins and apoptosis. Arch. Gynecol. Obstet..

[33] Doorbar J (2018). Host control of human papillomavirus infection and disease. Best Pract. Res. Clin. Obstet. Gynaecol..

[34] Pal A, Kundu R (2020). Human papillomavirus E6 and E7: The cervical cancer hallmarks and targets for therapy. Front. Microbiol..

[35] Kruchinin AA, Makarova AV (2023). Multifaceted nature of DNA polymerase θ. Int. J. Mol. Sci..

[36] Bubenik M, Mader P, Mochirian P, Vallée F, Clark J, Truchon J-F (2022). Identification of RP-6685, an orally bioavailable compound that inhibits the DNA polymerase activity of Polθ. J. Med. Chem..

[37] Cadoná FC, Machado AK, Bodenstein D, Rossoni C, Favarin FR, Ourique AF, Cadoná FC, Machado AK, Bodenstein D, Rossoni C, Favarin FR, Ourique AF (2020). Natural product–based nanomedicine: Polymeric nanoparticles as delivery cargoes of food bioactives and nutraceuticals for anticancer purposes. Advances and Avenues in the Development of Novel Carriers for Bioactives and Biological Agents.

[38] Sen P, Sahu PK, Haldar R, Sahu K, Prasad P, Roy A (2016). Apigenin naturally occurring flavonoids: Occurrence and bioactivity. Pharm. Biosci. J..

[39] Wang M, Firrman J, Liu L, Yam K (2019). A review on flavonoid apigenin: Dietary intake, ADME, antimicrobial effects, and interactions with human gut microbiota. BioMed. Res. Int..

[40] Xu L, Zaky MY, Yousuf W, Ullah A, Abdelbaset GR, Zhang Y (2021). The anticancer potential of apigenin via immunoregulation. Curr. Pharm. Des..

[41] Kim S, Chen J, Cheng T, Gindulyte A, He J, He S (2019). PubChem 2019 update: Improved access to chemical data. Nucleic Acids Res..

[42] Ferreira LG, Dos Santos RN, Oliva G, Andricopulo AD (2015). Molecular docking and structure-based drug design strategies. Molecules.

[43] Ravi L, Kannabiran K (2016). A handbook on protein-ligand docking tool: AutoDock 4. Innov. J. Med. Sci..

[44] Ohlenschläger O, Seiboth T, Zengerling H, Briese L, Marchanka A, Ramachandran R (2006). Solution structure of the partially folded high-risk human papilloma virus 45 oncoprotein E7. Oncogene.

[45] Liu X, Chen J, Wang Z, Wang D, He M, Qian C (2019). Neutralization sites of human papillomavirus-6 relate to virus attachment and entry phase in viral infection. Emerg. Microbes Infect..

[46] Eddershaw PJ, Beresford AP, Bayliss MK (2000). ADME/PK as part of a rational approach to drug discovery. Drug Discov. Today.

[47] Azzam KA (2023). SwissADME and pkCSM webservers predictors: An integrated online platform for accurate and comprehensive predictions for in silico ADME/T properties of artemisinin and its derivatives. Kompleksnoe Ispolzovanie Mineralnogo Syra.

[48] Gaviraghi G, Barnaby RJ, Pellegatti M, Testa B, van de Waterbeemd H, Folkers G, Guy R (2001). Pharmacokinetic challenges in lead optimization. Pharmacokinetic Optimization in Drug Research: Biological, Physicochemical, and Computational Strategies.

[49] Mortier J, Rakers C, Bermudez M, Murgueitio MS, Riniker S, Wolber G (2015). The impact of molecular dynamics on drug design: Applications for the characterization of ligand–macromolecule complexes. Drug Discov. Today.

[50] Lazim R, Suh D, Choi S (2020). Advances in molecular dynamics simulations and enhanced sampling methods for the study of protein systems. Int. J. Mol. Sci..

[51] Yalcin-Ozkat G (2021). Molecular modeling strategies of cancer multidrug resistance. Drug Resist. Updates.

[52] Murail S, Lacapere J-J (2017). Simulation of ligand binding to membrane proteins. Membrane Protein Structure and Function Characterization: Methods and Protocols.

[53] Joung IS, Cheatham TE (2008). Determination of alkali and halide monovalent ion parameters for use in explicitly solvated biomolecular simulations. J. Phys. Chem. B.

[54] Wells DB, Aksimentiev A (2010). Mechanical properties of a complete microtubule revealed through molecular dynamics simulation. Biophys. J..

[55] Nnyigide OS, Lee S-G, Hyun K (2018). Exploring the differences and similarities between urea and thermally driven denaturation of bovine serum albumin: Intermolecular forces and solvation preferences. J. Mol. Model..

[56] Rana N, Singh AK, Shuaib M, Gupta S, Habiballah MM, Alkhanani MF (2022). Drug resistance mechanism of m46i-mutation-induced saquinavir resistance in HIV-1 protease using molecular dynamics simulation and binding energy calculation. Viruses.

[57] Banerjee S, Majumder K, Gutierrez GJ, Gupta D, Mittal B (2020). Immuno-informatics approach for multi-epitope vaccine designing against SARS-CoV-2. BioRxiv.

[58] Cao W, Ding Z, Hang X, Xu K, Song J, Huang J (2018). Theoretical study of a series of 4, 4′-azo-1 H-1, 2, 4-triazol-5-one based nitrogen-rich salts as potential energetic compounds. RSC Adv..

[59] Rajalakshmi K, Gunasekaran S, Kumaresan S (2015). Density functional theory, comparative vibrational spectroscopic studies, highest occupied molecular orbital and lowest unoccupied molecular orbital analysis of Linezolid. Indian J. Phys..

[60] Kumer A, Sarker MN, Sunanda P (2019). The theoretical investigation of HOMO, LUMO, thermophysical properties and QSAR study of some aromatic carboxylic acids using HyperChem programming. Int. J. Chem. Technol..

[61] Mehmood A, Kaushik AC, Wang Q, Li C-D, Wei D-Q (2021). Bringing structural implications and deep learning-based drug identification for KRAS mutants. J. Chem. Inf. Model..

[62] Nath A, Kumer A, Zaben F, Khan M (2021). Investigating the binding affinity, molecular dynamics, and ADMET properties of 2, 3-dihydrobenzofuran derivatives as an inhibitor of fungi, bacteria, and virus protein. Beni-Suef Univ. J. Basic Appl. Sci..

[63] Shamsuddin T, Hosen MA, Alam MS, Emran TB, Kawsar SMA (2021). Uridine derivatives: Antifungal, PASS outcomes, ADME/T, drug-likeliness, molecular docking and binding energy calculations. Medicine.

[64] Siddikey F, Roni M, Kumer A, Chakma U, Matin M (2022). Computational investigation of Betalain derivatives as natural inhibitor against food borne bacteria. Curr. Chem. Lett..

[65] Humphrey W, Dalke A, Schulten K (1996). VMD: Visual molecular dynamics. J. Mol. Gr..

[66] Joshi AA, Narkhede SS, Viswanathan C (2005). Design, synthesis and evaluation of 5-substituted amino-2, 4-diamino-8-chloropyrimido-[4, 5-b] quinolines as novel antimalarials. Bioorg. Med. Chem. Lett..

[67] Qureshi R, Ghosh A, Yan H (2020). Correlated motions and dynamics in different domains of epidermal growth factor receptor with L858R and T790M mutations. IEEE/ACM Trans. Comput. Biol. Bioinf..

[68] Saha SK, Hens A, Murmu NC, Banerjee P (2016). A comparative density functional theory and molecular dynamics simulation studies of the corrosion inhibitory action of two novel N-heterocyclic organic compounds along with a few others over steel surface. J. Mol. Liq..

[69] Kumer A, Sarker MN, Paul S (2019). The thermo physical, HOMO, LUMO, vibrational spectroscopy and QSAR study of morphonium formate and acetate ionic liquid salts using computational method. Turk. Comput. Theor. Chem..

